# FeOOH Cocatalysts with Gradient Oxygen Vacancy Distribution Enabling Efficient and Stable BiVO_4_ Photoanodes

**DOI:** 10.1007/s40820-025-01987-8

**Published:** 2026-01-12

**Authors:** Shiyuan Wang, Mengjia Jiao, Qian Ye, Jie Jian, Fan Li, Guirong Su, Lu Zhang, Ziying Zhang, Zelin Ma, Jiulong Wang, Yazhou Shuang, Fang Wang, Yalong Song, Lichao Jia, Hongqiang Wang

**Affiliations:** 1 State Key Laboratory of Solidification Processing, Center for Nano Energy Materials, School of Materials Science and Engineering, Northwestern Polytechnical University and Shaanxi Joint Laboratory of Graphene (NPU), Shaanxi Laboratory for Advanced Materials, Northwestern Polytechnical University, Xi’an, 710072 People’s Republic of China; 2 School of Physics and Information Technology, Shaanxi Normal University, Xi’an, 710119 People’s Republic of China; 3 College of Materials Science and Engineering, Hohai University, Changzhou, 213200 People’s Republic of China; 4 School of Materials Science and Engineering, Shaanxi Normal University, Xi’an, 710119 People’s Republic of China; 5 International Research Center for Renewable Energy, Xi’an Jiaotong University, Xi’an, 710049 People’s Republic of China

**Keywords:** Photoetching, BiVO_4_ photoanodes, FeOOH cocatalysts, Oxygen vacancies, Photoelectrochemical water splitting

## Abstract

Highly active and stable FeOOH cocatalysts are essential for achieving optimal performance of BiVO_4_ (BVO) photoanodes. Despite offering remarkable structural stability, widely used thick FeOOH cocatalysts often suffer from insufficient hole transport capability, which hinders the overall activity. The present study demonstrates that a simple photoetching strategy is able to introduce gradient distributed oxygen vacancies (GO_V_) in the thick FeOOH layer and significantly enhances the photogenerated holes transport dynamics. The incorporation of GO_V_ within FeOOH not only realizes the “relay transport” of photogenerated hole through the progressive upward shift of the valence band in the spatial distribution, but also provides abundant oxidation active sites by efficient hole trapping. These improvements effectively improve the oxygen evolution reaction (OER) activities and mitigate photocorrosion by the instantaneous hole extraction. Consequently, the FeOOH-GO_V_ layer enables the BVO/FeOOH-GO_V_ photoanode to achieve an impressive photocurrent density of 5.37 mA cm^−2^ and a robust operational stability up to 160 h at 1.23 V_RHE_, setting new benchmarks for current density and stability in FeOOH-based BVO photoanodes. This work provides an effective avenue to optimize OER cocatalysts for constructing highly efficient and stable photoelectrochemical water splitting devices.

**Supplementary Information:**

The online version contains supplementary material available at 10.1007/s40820-025-01987-8.

## Highlights

- First demonstration of a gradient distributed oxygen vacancies (GO_V_) strategy to promote hole transport within FeOOH.
- Clearly monitoring and verifying the progressive upward shift of the valence band within the shallow surface of FeOOH-GOv for enhancing holes transport capability.
- Setting new photoelectrochemical activity and stability benchmarks of FeOOH based-BiVO_4_ photoanodes.

**Supplementary Information:**

The online version contains supplementary material available at 10.1007/s40820-025-01987-8.

## Graphical abstract

**Figure:**
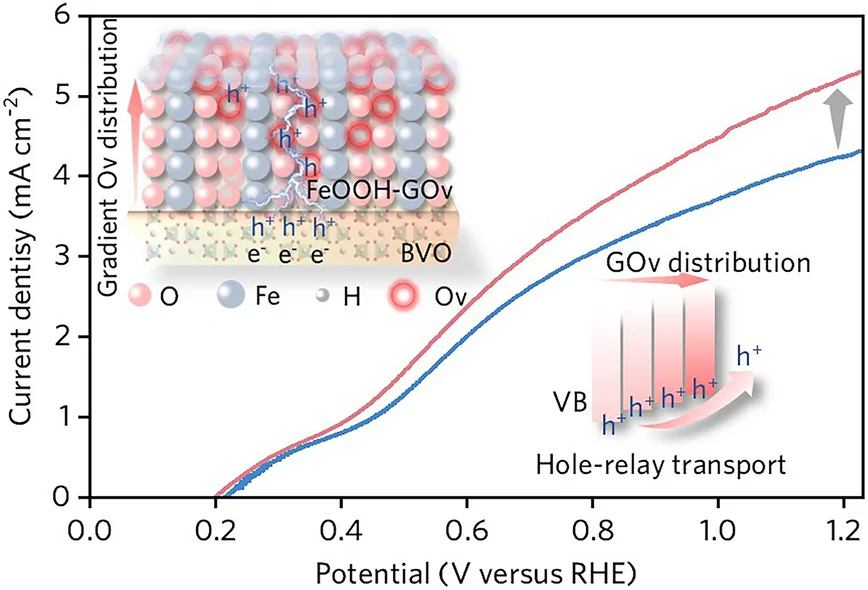

## Introduction

H_2_ production via photoelectrochemical (PEC) water splitting has been regarded as a promising approach for converting solar energy into sustainable clean fuel [1, 2]. The design of the suitable semiconductor photoanodes with efficient charge carrier transport and high surface oxidation reactivity is essential for practical PEC device [3, 4]. The bismuth vanadate (BiVO_4_, BVO) is notable among photoanode materials due to its narrow band gap (2.4 eV), suitable band edge positions, and high chemical stability. However, its solar energy conversion potential remains limited by severe carrier recombination, sluggish water oxidation kinetics, and susceptibility to photocorrosion [3, 5, 6]. Therefore, various modification strategies have been explored to overcome these challenges, including hetero-construction [7–10], nanostructuring [11], nanocrystal embedding [12], and defects engineering [13–16]. However, these methods have not yet fully resolved the sluggish water oxidation kinetics on the BVO surface, which is critical for achieving high solar conversion efficiency.

Coupling BVO with ultrathin iron oxyhydroxide (FeOOH) is a widely adopted strategy to enhance the PEC activity of BVO photoanodes [17–20]. As an oxygen evolution catalyst (OEC), FeOOH effectively extracts the photogenerated holes from the BVO surface, accelerating water oxidation with a reduced overpotential [18–21]. With the intensive efforts devoted to engineer FeOOH OEC in BVO photoanodes, such as crystalline phase control [19, 20], defects modulating [19, 22, 23], and heterogeneous atom doping [24, 25], the photocurrent density has reached a notable benchmark value as high as 5.2 mA cm^−2^ at 1.23 V_RHE_ [19], while this high PEC activity of BVO photoanodes coupled with ultrathin FeOOH is unfortunately obtained at the expense of stability. This limited stability is primarily attributed to the generally employed ultrathin FeOOH layer that is intrinsically favors for holes transport but with the awkward feature of structural instability at high anodic potential (Fig. S1A). Therefore, creating efficient and stable BVO photoanodes requires precise control over the coupled OEC thickness for a better balance of the oxygen evolution reaction (OER) activity and durability [26, 27]. A recent study revealed that increasing OEC layer thickness to over 10 nm resulted in pronounced stability of BVO compared to the mostly adopted ultrathin layer (3–5 nm) [27]. However, the insufficient hole transport capability of the thick FeOOH OEC layer would no doubt impair the ideal OER kinetics (Fig. S1B) [23, 28, 29]. Therefore, it is highly desirable but challenging to develop efficient FeOOH OECs that are featured with simultaneously activated hole transport kinetics and improved PEC stability.

**Scheme 1:**
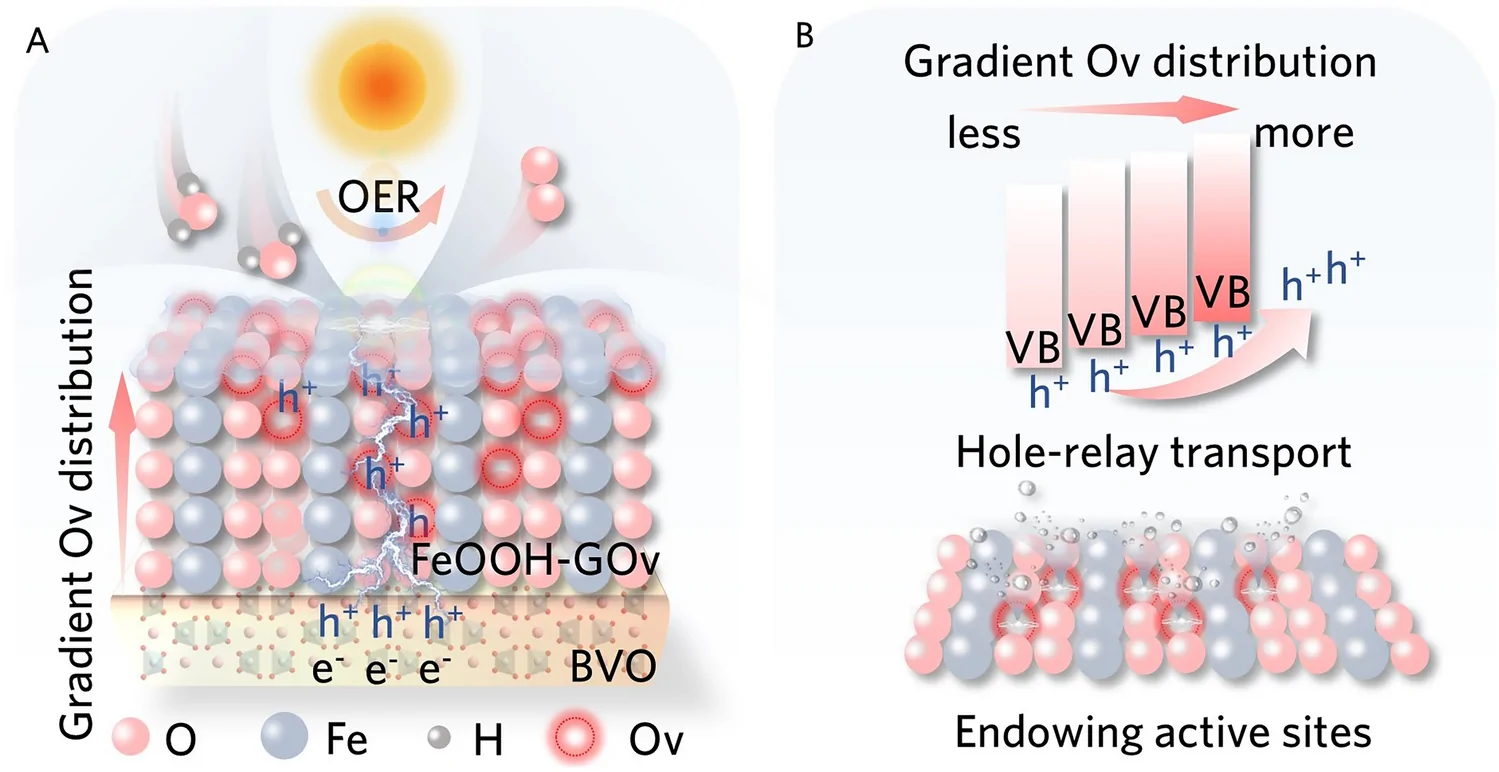
**A** Illustration of the insufficient hole transport capability and great structure stability of thick FeOOH layer decorated on BVO photoanodes. **B** Schematic illustration of the boosted PEC performance of FeOOH-GO_V_ OECs

Presented is a facile but effective strategy of gradient distributed oxygen vacancy (GO_V_) engineering via a simple photoetching (PE) treatment applied to a thick coupled FeOOH layer, which enables activating hole transport kinetics of FeOOH, thus comprehensively boosting the PEC activity and stability of BVO photoanodes (Scheme 1A). During this PE process, BVO/FeOOH photoanode was immersed in a potassium phosphate buffer (KPi) with sodium sulfite (Na_2_SO_3_) as an additive, which significantly accelerated GO_V_ generation through rapid photoreduction. The substantial incorporation of GO_V_ facilitates the hole transport by the progressive upward shift of the valence band maximum (VBM) within the spatial distribution, exhibiting characteristic “relay transport” behavior (Scheme 1B), significantly increasing the charge injection efficiency (*η*_inj_) up to 98.88% (1.23V_RHE_). Furthermore, the high-density surface Ov induced by GO_V_ incorporation endows plenty of oxidation active sites via effective hole trapping, thus improving the PEC activity (Scheme 1B). On the basis of the inherent good stability in thick FeOOH, the faster holes extraction effectively suppresses the photocorrosion thus further improving the operational stability. As a result, the construction of FeOOH-GO_V_ layer increases the photocurrent density of BVO/FeOOH-GO_V_ photoanode to 5.37 mA cm^−2^, with enhanced operational stability up to 160 h at 1.23 V_RHE_. These values are among the top both in the current density and stability records of FeOOH-based BVO photoanodes (Table S1). As such, present study provides an effective strategy to overcome the challenge of poor carrier transport kinetics in thick OECs, paving the way for efficient and stable photoelectrodes toward practical application.

## Experimental Section

### Preparation of the BVO/FeOOH-GOV Films

The BVO photoanodes were fabricated via an electrochemical deposition method. Subsequently, the prepared BVO photoanodes were subjected to a chemical bath treatment in a 10 mL of 0.01 M FeCl_3_ solution within a binary solvent system (H_2_O-DMSO = 3:1, v/v) for 10 h at 50 °C to obtain BVO/FeOOH films. Finally, the as-prepared BVO/FeOOH films were immersed in the 1 M KPi buffer solution with 0.1 M Na_2_SO_3_ for different times under illumination by A Xe 500 W lamp (AM 1.5G). Detailed experimental procedures for the fabrication of BVO/FeOOH-GO_V_ photoanodes are described in the Supporting Information file.

### Material Characterization

All BVO/FeOOH-GO_V_ samples were comprehensively characterized and analyzed using a series of analytical techniques, including the field emission scanning electron microscopy (FESEM), X-ray diffraction (XRD), ultraviolet–visible (UV–vis) spectra, transmission electron microscopy (TEM), transmission electron microscopy energy-dispersive spectroscopy (TEM-EDS, Raman spectroscopy, X-ray photoelectron spectroscopy (XPS), photoluminescence (PL) spectra, femtosecond pump-probe transient absorption spectroscopy (TAS), the scanning transmission electron microscopy coupled with electron energy-loss spectroscopy (STEM-EELS), electron paramagnetic resonance (EPR), and intensity modulated photocurrent spectroscopy (IMPS), respectively. Detailed experimental parameters for each analytical method are provided in the Supporting Information file.

### Photoelectrochemical Characterization

The photoelectrochemical measurements of all BVO/FeOOH samples were conducted using a standard three-electrode configuration (CHI660E electrochemical workstation) under ambient conditions. The working electrode with 0.25 cm^2^ active area was prepared for each sample, with Pt and Ag/AgCl (saturated KCl) serving as counter and reference electrodes, respectively. 1.0 M potassium phosphate buffer (pH = 7) were used as the electrolyte. Illumination was provided by a 500 W lamp (CEL-S500, CEAULIGHT) equipped with an AM 1.5G filter to simulate standard solar irradiance (100 mW cm^−2^ at electrode surface). All photoelectrode samples for photoelectrochemical measurements were illuminated from backside. Detailed measurement protocols and parameter calculations are comprehensively documented in the Supporting Information file.

## Results and Discussion

### Morphology and Structure Characterizations

**Fig. 1:**
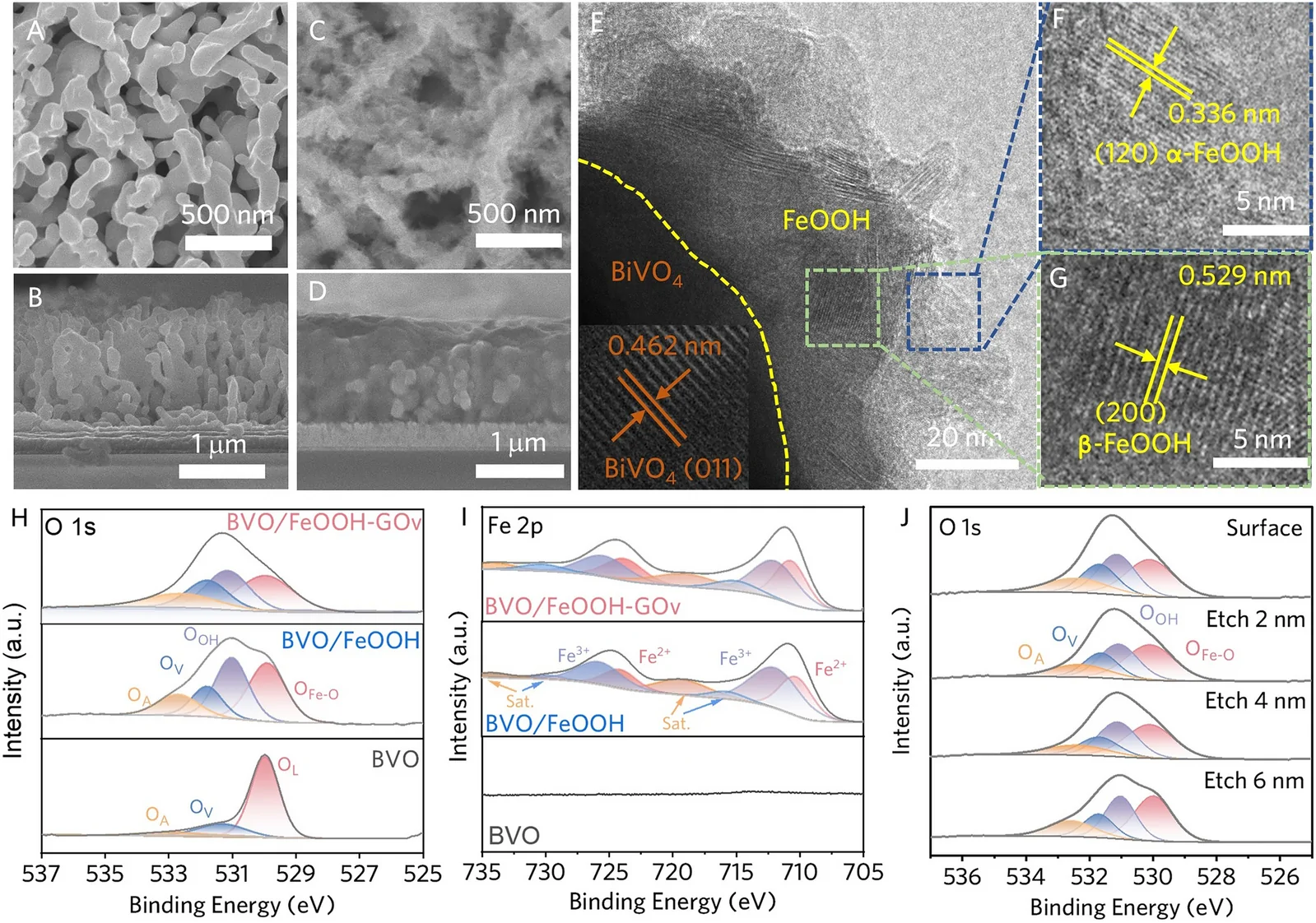
**A** SEM image and **B** cross-sectional view of SEM image of bare BVO. **C** SEM image and **D** cross-sectional view of SEM image of BVO/FeOOH-GO_V_. **E** TEM image of BVO/FeOOH-GO_V_ film (insert: HRTEM image of BVO). HRTEM images of **F** area labeled in bule and **G** area labeled in green in **D**. **H** XPS of O 1*s* of bare BVO, BVO/FeOOH and BVO/FeOOH-GO_V_ films. **I** XPS of Fe 2*p* peaks of bare BVO, BVO/FeOOH, and BVO/FeOOH-GO_V_ films. **J** Depth profiles of O 1*s* for BVO/FeOOH-GO_V_ film

Figure S2A depicts the fabrication process of the BVO/FeOOH photoanode. First, a nanoporous BVO film was synthesized by an electrodeposition method [18]. Then, the FeOOH layer as OEC was loaded onto the surface of BVO photoanode via a chemical bath deposition, resulting in a BVO/FeOOH photoanode. Subsequently, the as-prepared BVO/FeOOH photoanode underwent a PE process to obtain the photoetched FeOOH decorated BVO photoanode (Fig. S2B, denoted as BVO/FeOOH-GO_V_). This process involved immersing the BVO/FeOOH photoanode in 1 M KPi electrolyte containing 0.1 M Na_2_SO_3_ under 5 h of illumination (AM 1.5G). The bare BVO photoanode initially exhibits smooth surface formed by nanoporous particles with a size of ~ 200 nm and a thickness of ~ 1.5 μm (Fig. 1A, B). Upon loading with FeOOH-GO_V_, the BVO surface evolves into a rough flocculent structure while maintaining the original particle size and thickness (Fig. 1C, D), which is similar to the BVO/FeOOH photoanode (Fig. S3).

The structure, thickness, and coverage of FeOOH-GO_V_ on the BVO surface were further illustrated by XRD patterns and TEM images, respectively. Compared with the bare BVO photoanode, distinct peaks corresponding to *α*-FeOOH (JCPDF No. 29–0713) and *β*-FeOOH (JCPDF No. 34–1266) are observed both in the XRD patterns of BVO/FeOOH and BVO/FeOOH-GO_V_ films, respectively (Fig. S4), demonstrating that the FeOOH is present in a mixed-phase structure. There is no evident peak change in the XRD patterns between BVO/FeOOH and BVO/FeOOH-GO_V_ films, indicating that the crystal structure of the BVO/FeOOH film does not change after PE treatment. Figure 1E shows a uniform and fully covered thick layer with thickness of ~ 40 nm on the BVO surface, compared to the bare BVO film (Fig. S5). The HRTEM images (Figs. 1E insert, 1F and 1G) show an interlayer spacing of 0.462 nm for the (011) planes of BiVO_4_, 0.336 nm for the (120) crystal planes of *α*-FeOOH and 0.529 nm the (200) crystal planes of *β*-FeOOH, respectively, confirming the mixed-phase structure of FeOOH-GO_V_ on BVO. The mixed phase is attributed to the unique property of DMSO, which accelerates the hydrolysis reaction, thus leading to the two phases coexisting at an appropriate hydrolysis rate [17]. The mixed-phase structure also exists in BVO/FeOOH film (Fig. S6). The TEM-EDS images (Fig. S7) confirm the homogeneous FeOOH-GO_V_ coverage on BVO film, which enhances visible light responsiveness and narrows the bandgap of the BVO film (Fig. S8).

**Fig. 2:**
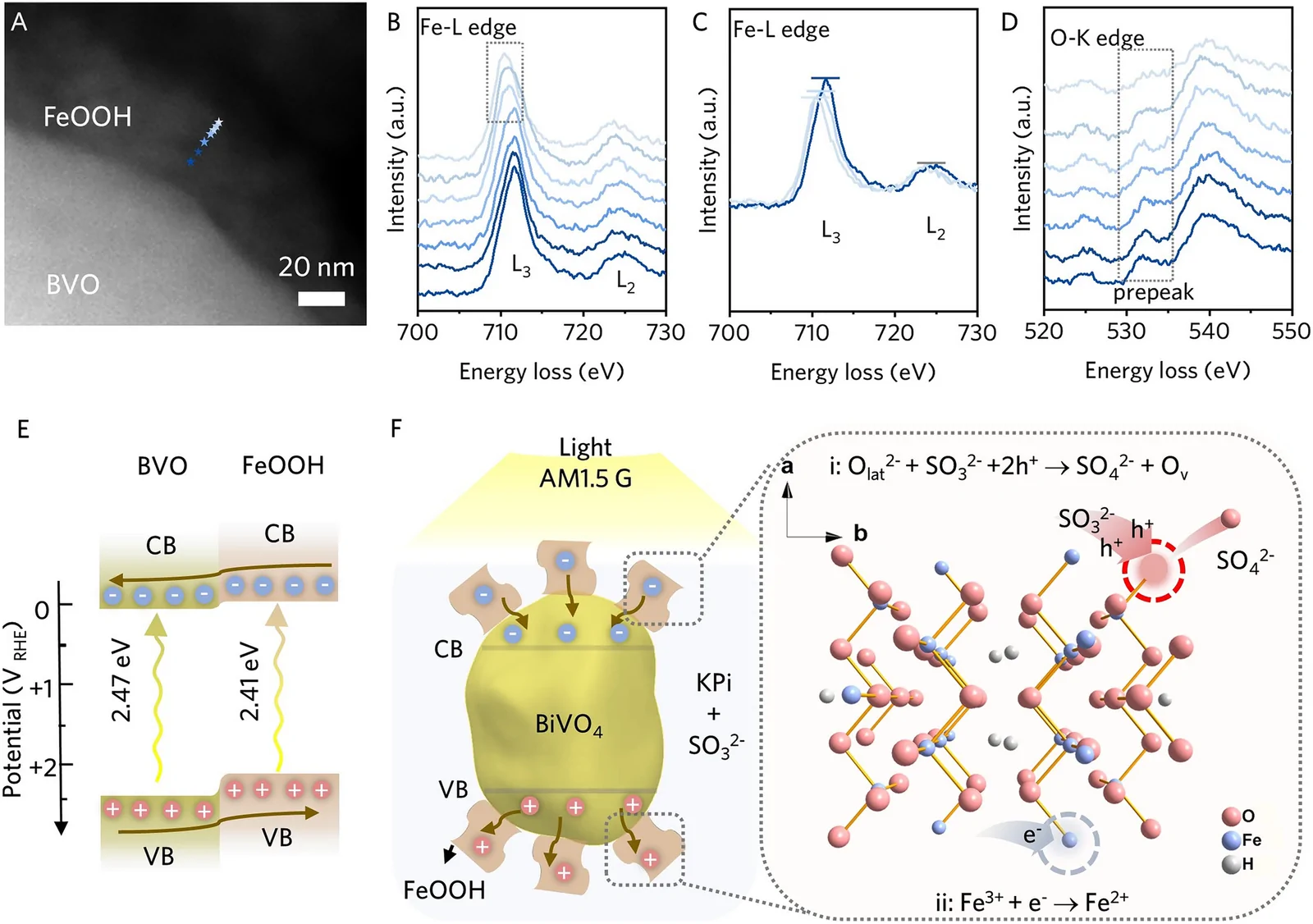
**A** STEM image of BVO/FeOOH-GO_V_ film with the probing path, **B** the corresponding EELS spectra of Fe L_2,3_ edge with the probing path of **A**, **C** the selected EELS spectra of Fe L_2,3_ edge, and **D** the corresponding EELS spectra of O K edge with the probing path of **A**. **E** Band alignment structure between BVO and FeOOH. **F** Illustration of the generation of GO_V_ in FeOOH during the PE treatment process in KPi with 0.1 M Na_2_SO_3_

XPS measurements were carried out to explore the impacts of PE treatment on electronic structure of FeOOH. The O 1*s* XPS spectra of BVO, BVO/FeOOH, and BVO/FeOOH-GO_V_ films (Fig. 1H) can be deconvoluted into four peaks corresponding to lattice oxygen (O_Fe-O_, 529.90), lattice OH group (O_OH_, 531.08), oxygen vacancy (O_V_, 531.76), and adsorbed oxygen (O_A_, 532.57), respectively [19, 24, 30–33]. Notably, the O_V_ ratio increases from 15.54% to 19.99% after PE treatment (Fig. 1H and Table S2), preliminarily proving that PE treatment introduces O_V_ at the FeOOH surface. In addition, the XPS spectra of Fe 2*p* (Fig. 1I, Table S3) and EPR spectra (Fig. S9) further support the existence of O_V_ in BVO/FeOOH-GO_V_ films [31]. To investigate the distribution of O_V_ in BVO/FeOOH-GO_V_ films, the XPS depth profile characterization of O 1* s* signal was performed. The results indicate a gradient distribution of O_V_, decreasing from 19.99% at the surface to 15.59%, in the shallow surface region (approximately 6 nm) of FeOOH (Fig. 1J and Table S4). Besides, SS etching depth increases, the decreasing proportion of Fe^2+^ and the negative binding energy shift of the Bi 4*f*/V 2*p* peaks further confirm the GO_V_ distribution in FeOOH (Fig. S10). To further confirm the GO_V_ distribution of FeOOH, the STEM-EELS was conducted on the BVO/FeOOH-GO_V_ film. Figure 2A displays the STEM image of the BVO/FeOOH-GO_V_ film, with the blue star line indicating the EELS probe path. Analysis of the Fe L_2,3_ edges reveals a distinct energy shift in the L_3_ peak toward lower loss values (Fig. 2B) and a concurrent decrease in the L_3_/L_2_ white-line ratio (Fig. 2C) as the probe approaches the FeOOH surface (within a depth of ~ 6 nm). These trends signify a progressive reduction in the average iron oxidation state within a depth of ~ 6 nm on the surface [34–36], which is directly correlates with GO_V_ formation. The O-K edge spectra (Fig. 2D) provide complementary evidence, where the pre-edge peak intensity attenuates markedly within the same 6 nm near-surface region, directly supporting the existence of GO_V_ [37, 38]. The above analysis demonstrates that the FeOOH structure retains its structural integrity despite the introduction of GO_V_.

### Mechanism for the Generation of GO_V_ in FeOOH

It is found that the effectiveness of GO_V_ generation in FeOOH during PE treatment process is significantly reduced in the absence of Na_2_SO_3_ (Fig. S11A, B, H, and Discussion S1), indicating Na_2_SO_3_ is essential for GO_V_ formation in FeOOH during PE treatment process. Therefore, a possible mechanism is proposed for the generation of GO_V_ in FeOOH (Fig. 2E, F). First, bandgap illumination generates electron–hole pairs within BVO and FeOOH, respectively [39, 40]. Second, owing to the established Type II heterojunction (n–n junction) between BVO and FeOOH (Figs. 2E, S12, and Table S5), the photogenerated holes in the VBM of BVO spontaneously migrate toward that of FeOOH, while the photogenerated electrons in the CBM of FeOOH concurrently transfer to BVO (Fig. 2E, F) [41]. Then, some of the holes stored in FeOOH directly react with SO_3_^2−^ and lattice oxygen under illumination, leading to the formation of O_V_ and SO_4_^2−^, while some of the electrons in FeOOH which are going to be transferred to BVO, quickly react with lattice Fe^3+^ions to form Fe^2+^ defective sites before reaching BVO due to charge conservation (Fig. 2F, Eqs. i–ii). The reaction process of FeOOH photoetched in KPi without Na_2_SO_3_ is illustrated in Fig. S11C, where the holes oxidize O_lat_^2−^ and release O_2_, leaving O_V_ on the FeOOH surface. This oxidation reaction is a four-electron reaction process that requires not only the gradual participation of 4 holes (Fig. S11C, Eqs. i–ii), but also the cooperation of neighboring O_lat_^2−^ to release an O_2_, therefore resulting in a sluggish kinetics [42]. In contrast, the reaction involving SO_3_^2−^ only needs two holes and does not depend on the neighboring O_lat_^2−^, leading to a fast kinetics and thus enhancing the PE reaction efficiency under the same illumination time. Consequently, the surface photoetching reaction is more pronounced under the combined action of illumination and electrolyte, thus resulting in GO_V_ within FeOOH.

In order to further verify the effectiveness of the photogenerated electrons in reducing Fe^3+^ during the PE process, the same experiment under different applied biases were performed (Fig. S13A–E, and Discussion S2). The J-V curves of the samples after photoelectric etching (PEE) are shown in Fig. S13A–D. When the applied bias exceeds the flat-band potential, photogenerated electrons are effectively extracted and transferred to the counter electrode, and the photocurrent density of the etched sample decreases significantly with increasing applied anode bias during the PEE process (Fig. S13B–D). At the same time, the XPS Fe 2*p* analyses of the three samples after PEE treatment revealed an inverse correlation between applied bias and the proportion of Fe^2+^: higher applied biases resulted in lower Fe^2+^ content (Fig. S14A–C), indicating a corresponding decrease in O_V_ concentration. This indicates that the photogenerated electrons in BVO/FeOOH play a key role in the etching process. Applying a sufficient positive bias effectively diverts these electrons to the counter electrode, thereby preventing their participation in the reduction of Fe^3+^ in FeOOH (Fe^3+^ + e^⁻^ → Fe^2+^), confirming the necessity of the electron-mediated Fe^3+^ reduction step for the oxidation of lattice oxygen and subsequent O_V_ formation. Conversely, under photoetching conditions without applied bias, while the band alignment thermodynamically favors electron transfer from FeOOH to BVO, the weaker driving force for electron extraction compared to the applied anode bias allows photogenerated electrons in FeOOH which are going to be transferred to BVO to be rapidly consumed (Fe^3+^  + e⁻ → Fe^2+^) before reaching BVO by the charge compensation reaction. In addition, in a SO_3_^2−^-free PEE system, the photocurrent density of the etched sample remained largely unchanged (Fig. S13E), and the Fe^2+^ proportion showed minimal variation compared to pure BVO/FeOOH (Fig. S14D). These results further strongly support the dominant role of SO_3_^2−^ in driving O_V_ formation through lattice oxygen oxidation. On the basis of this evidence, the origin of the GO_V_ distribution in FeOOH is established.

### PEC Performance of the BVO/FeOOH-GO_V_

**Fig. 3:**
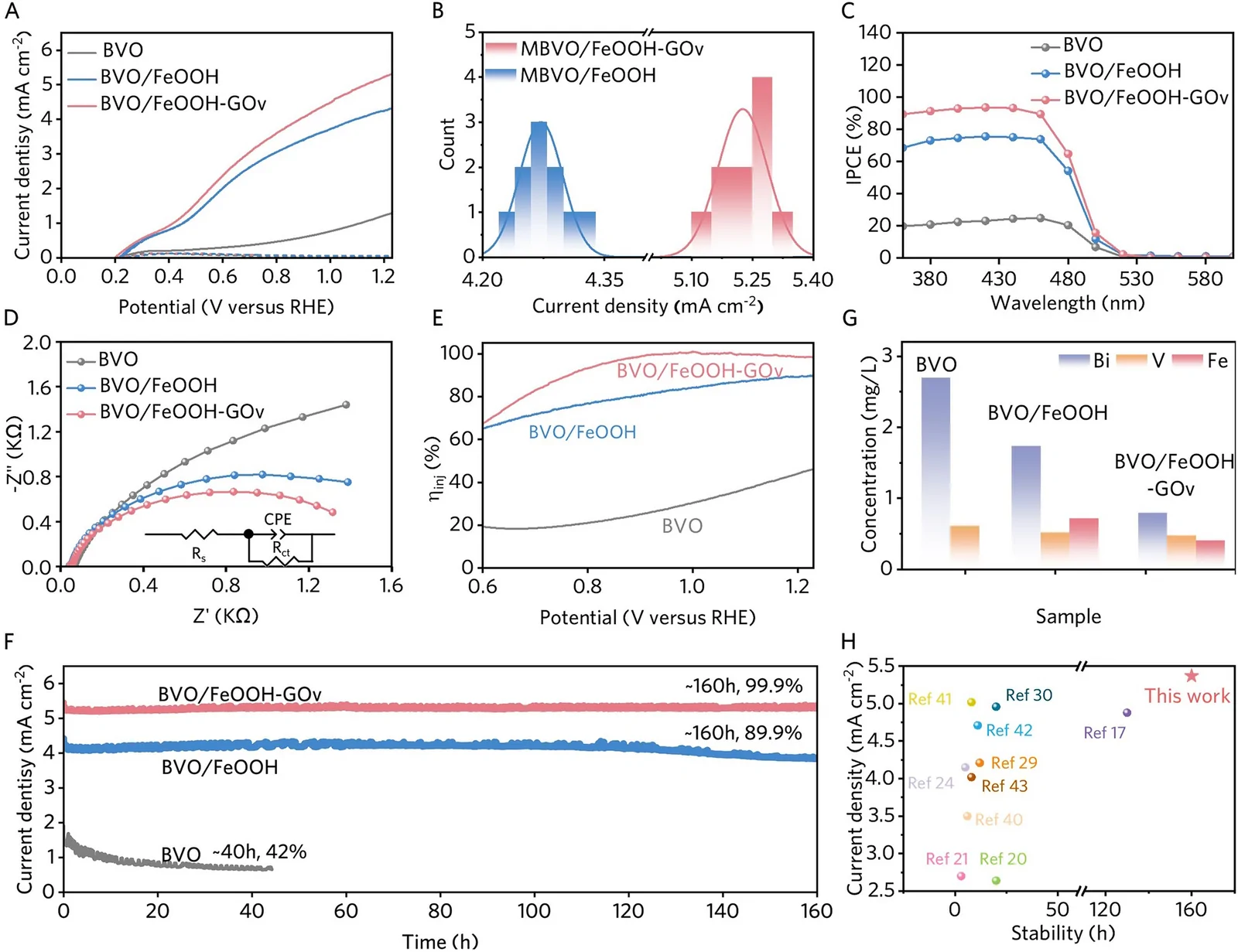
**A** J-V curves of bare BVO, BVO/FeOOH and BVO/FeOOH-GO_V_ films measured in 1 M potassium phosphate buffer (pH = 7, the dash line is the corresponding dark current). **B** Photocurrent density distribution of BVO/FeOOH-GO_V_ and BVO/FeOOH films at 1.23 V_RHE_ measured in 1 M potassium phosphate buffer (pH = 7). **C** IPCEs, **D** EIS curves and **E** charge injection efficiencies of bare BVO, BVO/FeOOH, and BVO/FeOOH-GO_V_ films measured in 1 M potassium phosphate buffer (pH = 7). **F** Long-term stability of the bare BVO, BVO/FeOOH, and BVO/FeOOH-GO_V_ photoanodes at 1.23 V_RHE_ measured in 1 M potassium phosphate buffer (pH = 7). **G** ICP analyses of different KPi after stability measurement. **H** Comparison of the current density and stability of BVO/FeOOH-GO_V_ photoanode with the previously reported FeOOH-related BVO photoanodes

The PEC water splitting performances of all samples were evaluated in 1.0 M KPi (pH = 7) using a standard three-electrode cell system under backside AM 1.5 G illumination (100 mW cm^−2^). The bare BVO exhibits a low photocurrent of 1.28 mA cm^−2^ at 1.23 V_RHE_, while the photocurrent increases significantly up to 4.31 mA cm^−2^ after the decoration of FeOOH (Fig. 3A), indicating that the oxygen evolution activity is promoted by the FeOOH cocatalyst. Further optimization of the PE time for BVO/FeOOH films results in an outstanding photocurrent density of 5.37 mA cm^−2^ at 1.23 V_RHE_ for BVO/FeOOH-GO_V_ film (Figs. 3A and S15 (photoetching treatment for 5 h)). The photocurrent density histograms of 10 samples for BVO/FeOOH and BVO/FeOOH-GO_V_, respectively, confirm the pronounced effects of GO_V_ within FeOOH on improving PEC performance (Fig. 3B). The maximum applied bias photon-to-current efficiency (ABPE) for the BVO/FeOOH-GO_V_ reaches 1.61% at 0.7 V_RHE_, compared to 1.38% for BVO/FeOOH at 0.7 V_RHE_ and only 0.20% for BVO at 0.82 V_RHE_, respectively (Fig. S16A). The incorporation of GO_V_ also significantly enhances incident-photon-to-current conversion efficiency (IPCE) values compared to the bare BVO and BVO/FeOOH films over the entire wavelength range (Fig. 3C). Especially at 440 nm, the IPCE of the BVO/FeOOH-GO_V_ sample reaches up to 93.65%. By integration of IPCE values, the calculated photocurrent densities closely match the measured values of 1.28, 4.31, and 5.37 mA cm^−2^ at 1.23 V_RHE_ from the J-V curves for BVO, BVO/FeOOH, and BVO/FeOOH-GO_V_ photoanodes (Figs. S17 and 3A), demonstrating the reliability of the photocurrent densities obtained in the J-V curves. Additionally, the absorbed photon-to-current efficiency (APCE) of the BVO/FeOOH-GO_V_ photoanode reaches 98.29% at 440 nm, which is much higher than BVO/FeOOH (82.74%) and BVO (29.88%) (Fig. S16B).

Except for the high conversion efficiencies, the favorable interfacial charge transfer characteristics were further confirmed by the electrochemical impedance spectroscopy (EIS). According to the Nyquist plots and the fitting results (Fig. 3D, and Table S6), the BVO/FeOOH-GO_V_ photoanode exhibits significantly lower charge transfer resistance (R_ct_, 826 Ω) compared with BVO/FeOOH (1054 Ω) and bare BVO (6645 Ω), revealing improved interface charge transfer behavior between BVO and FeOOH-GO_V_. To obtain in-depth insights of the charge separation and transport, Mott–Schottky (M-S) curves of BVO, BVO/FeOOH, and BVO/FeOOH-GO_V_ films were conducted. The M-S plots reveal that FeOOH-GO_V_ significantly improves the charge carrier density (N_D_) from 5.78 × 10^18^ (BVO/FeOOH) to 8.60 × 10^18^ (BVO/FeOOH-GO_V_)), while negatively shifts the flat-band potential (E_fb_) from 0.30 V_RHE_ (BVO/FeOOH) to 0.28 V_RHE_ (BVO/FeOOH-GO_V_) (Fig. S18A, and Table S7), resulting in a superior charge transfer efficiency of BVO/FeOOH-GO_V_ photoanode. This was consistent with the calculated *η*_sep_ results of these samples (Fig. S18B). Besides, the charge injection efficiency (*η*_inj_) at 1.23 V_RHE_ of BVO/FeOOH-GO_V_ photoanode reaches 98.88%, significantly higher than that of the bare BVO photoanode (45.77%) and BVO/FeOOH (90.12%) (Fig. 3E), confirming the contribution of GO_V_ to the improvement of photogenerated hole transfer within FeOOH layer.

To investigate the durability of BVO films with uniform FeOOH decoration, the long-term stability test for the as-prepared samples were recorded at 1.23 V_RHE_ in 1.0 M KPi electrolyte. As shown in Fig. 3F, the photocurrent density of the bare BVO film drops from to 1.28 to 0.53 mA cm^−2^ at 1.23 V_RHE_ (dropping to 42%) after 40 h, which should be ascribed to the severe anodic photocorrosion [7, 26, 43, 44]. This is further supported by the inductively coupled plasma optical emission spectrometry (ICP-OES) analyses of the electrolyte used in the stability tests (Fig. 3G). After the decoration of FeOOH, the dissolution of V^5+^ and Bi^3+^ is relieved, the photocurrent density of the BVO/FeOOH film drops from 4.31 to 3.83 mA cm^−2^ at 1.23 V_RHE_ (dropping to 89.9%) after 160 h. Remarkably, the BVO/FeOOH-GO_V_ photoanode maintains stability for a total of 160 h (99.9%) at 1.23 V_RHE_ without a significant degradation (dropping from 5.37 to 5.31 mA cm^−2^ at 1.23 V_RHE_). The ICP-OES analyses for the electrolyte used for BVO/FeOOH-GO_V_ film indicate that a marked reduction in the dissolution of V and Bi cations, as well as a decrease in Fe concentration. The stability improvement is attributed to the GO_V_ acting as the stable hole transfer agent in FeOOH, which mitigates photocorrosion and finally improves stability. With a comprehensive comparison of the PEC performance for FeOOH-based BVO photoanodes, the photocurrent density and stability obtained in this work significantly surpass those of O_V_ modified FeOOH-based BVO photoanodes, as well as all of FeOOH-based BVO photoanodes (Tables S1,S8, and Fig. 3H) [20, 21, 24, 29, 30, 45–48].

### Effect of Photoinduced GO_V_ in FeOOH on the Boosted Carrier Transport Behavior

**Fig. 4:**
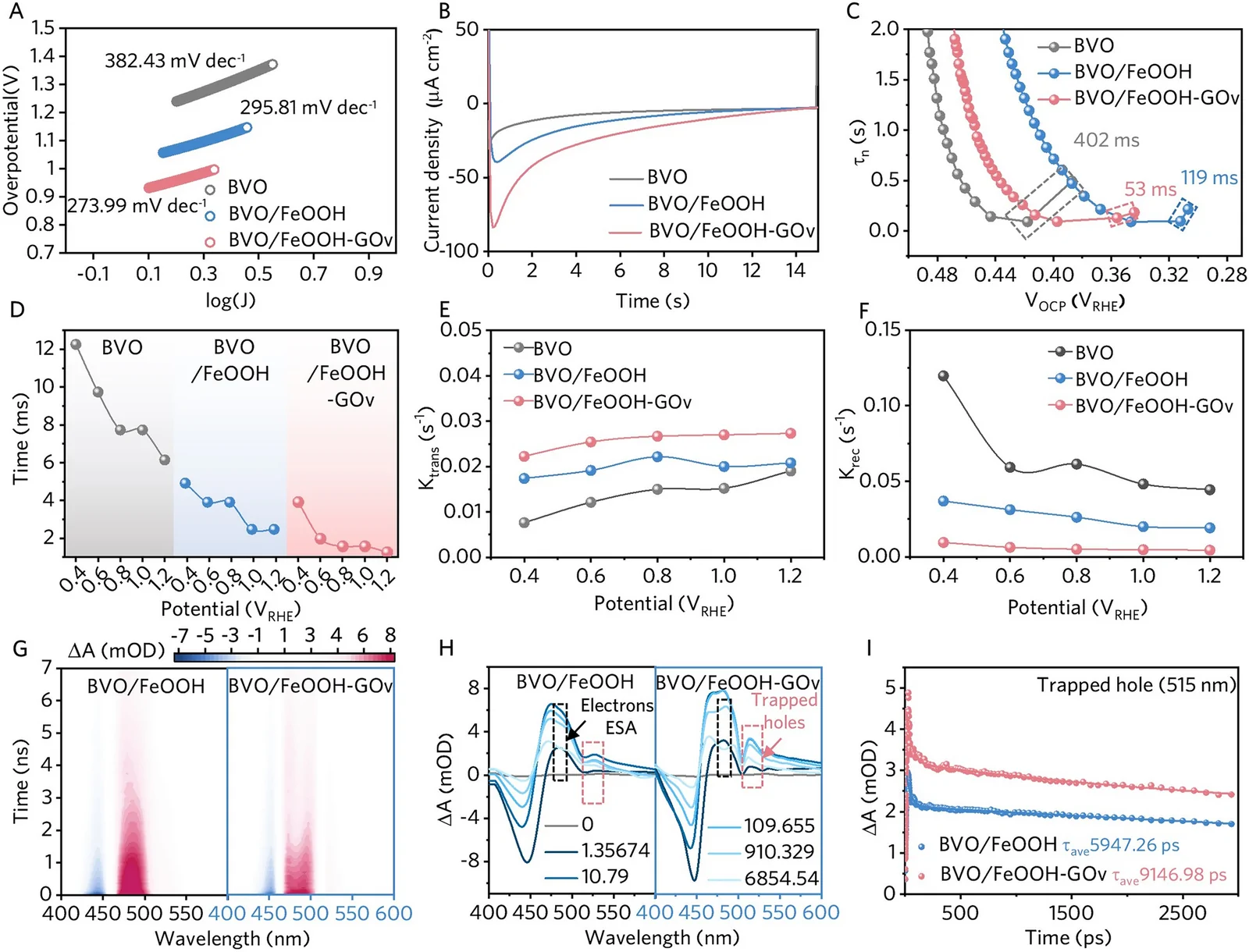
**A** Tafel slope curves, **B** Delay of the cathodic photocurrent curves measured at 0.4 V_RHE_ under dark condition, **C** OCP-derived carrier transfer lifetimes, **D** Carriers transport time, **E** K_trans_ and **F*** K*_rec_ of BVO, BVO/FeOOH, and BVO/FeOOH-GO_V_ films **G** TA spectra, **H** TAS at selected delay times **I** Corresponding TAS decay profiles of trapped holes signals of BVO/FeOOH and BVO/FeOOH-GO_V_ films, respectively

To clarify the improved water oxidation activity and boosted charge carrier transport behavior of BVO film decorated with FeOOH-GO_V_, a series of electrochemical and spectrum analyses were performed on BVO/FeOOH-GO_V_, BVO/FeOOH, and BVO photoanodes. Figure S19 shows C-V curves and the Δcurrent density scan rate curves of three films, which are used to estimate the electrochemical active area of these photoanodes. The double-layer capacitance of BVO, BVO/FeOOH, and BVO/FeOOH-GO_V_ photoanodes are 157, 277, and 303 μF cm^−2^, respectively. This indicates that the BVO/FeOOH-GO_V_ photoanode carries the largest electrochemical active area which can provide the most active sites for photoelectrochemical reactions. Furthermore, compared with the BVO/FeOOH and bare BVO, the BVO/FeOOH-GO_V_ photoanode shows an earlier electrocatalytic onset shift of 150–180 mV (Fig. S20). Meanwhile, the estimated Tafel slope of the BVO/FeOOH-GO_V_ photoanode (273.99 mV dec^−1^) is much smaller than those of the BVO/FeOOH (295.81 mV dec^−1^) and BVO (382.43 mV dec^−1^) photoanodes (Fig. 4A). Such earlier electrocatalytic onset and smaller Tafel slope for BVO/FeOOH-GO_V_ photoanode both demonstrate that the water oxidation reaction kinetic of FeOOH-GO_V_ is superior to that of FeOOH cocatalyst.

The hole extraction capability of the BVO/FeOOH-GO_V_ photoanode was quantified by measuring the number of holes stored against an applied bias, which can be calculated from the delay in the steady-state cathodic current based on the transient state photocurrent curves measured under chopped illumination [49]. The delay of the cathode current indicates that the photogenerated holes reaching the electrode/electrolyte interface do not participate in water oxidation, but are instead stored at the electrode surface, which thus can effectively reflect the hole extraction capability of photoelectrode [49]. As shown in Figs. 4B and S21, the number of holes stored at 0.4 V_RHE_ for the BVO/FeOOH-GO_V_ photoanode is obviously higher than that of BVO/FeOOH and BVO photoanode, indicative of the presence of long-lived holes at the surface of BVO/FeOOH-GO_V_ (Discussion S3), thus demonstrating the strong hole extraction capability of the FeOOH-GO_V_ for water oxidation. Open circuit potential (OCP) analyses offer valuable information on the built-in electric field of photoanodes and its impact on the photogenerated carrier behavior. The larger photovoltage (ΔOCP = OCP_dark_—OCP_light_) generated in BVO/FeOOH-GO_V_ film suggests that the FeOOH-GO_V_ exhibits more efficient capability than normal FeOOH for providing larger driving force to inject the photogenerated holes into the electrolyte during the PEC reaction (Fig. S22). The carrier lifetime (τ_n_) derived from the OCP transient decay profile, further elucidates the charge transport behavior at the photoanode/electrolyte junction [50, 51]. As shown in Fig. 4C, the BVO/FeOOH-GO_V_ displays a *τ*_n_ of 53 ms at the transient when the illumination is stopped, which shows four times lower than that of BVO/FeOOH (119 ms) and one order of magnitude lower than that of bare BVO (402 ms), indicative of a markedly improved carrier transfer behavior of the BVO/FeOOH-GO_V_ photoanode under illumination [51–53].

The IMPS was employed to investigate the charge transfer and surface recombination kinetics of various photoanodes. The typical IMPS responses of these three photoanodes measured at potential range of 0.4–1.23 V_RHE_ are shown in Fig. S23A–C. By extracting the parameters from the IMPS plots of these samples (Fig. S23), the transfer time (*τ*_d_) of the photo-induced carriers, charge transfer (*K*_trans_), and recombination (*K*_rec_) rate constants can be obtained, respectively [52, 54, 55]. The lowest *τ*_d_ and *K*_trans_ values of BVO/FeOOH-GO_V_ photoanode over the entire measured potential range (Fig. 4D–F, and Discussion S4) indicates that GO_V_ plays significant roles in facilitating charge carrier transfer from the bulk material to the surface [56]. In contrast, it is evident that the deposition of FeOOH on the BVO photoanode effectively reduces K_rec​_ at the same applied potential and eliminates the non-monotonic trend of *K*_rec_ for BVO film. This reduction can be primarily attributed to the ability of FeOOH to passivate the surface states of BVO. Introducing GO_V_ further reduces the *K*_rec_ in BVO/FeOOH-GO_V_ and its voltage dependence remains monotonic and similar to unmodified BVO/FeOOH. These suggest that the O_V_ concentration changes caused by GO_V_ distribution facilitate the transport of photogenerated holes within FeOOH. As a result, more photogenerated holes migrate to the catalyst surface, where they are rapidly consumed, leading to a significant suppression of charge carrier recombination. This reduced carrier recombination kinetics is also supported by PL measurement (Fig. S24, and Discussion S5). These results demonstrate that the incorporation of GO_V_ into FeOOH effectively suppresses interfacial recombination and enhances carrier transfer from BVO to the catalyst surface, thereby significantly improving water oxidation activity.

TAS was performed to investigate the photogenerated charge carrier dynamics, which makes it possible to monitor the variation of the species, as well as the fates of photogenerated holes and electrons on the timescale of ps-μs [52, 57]. After the deposition of FeOOH, the ground state bleaching (GSB) peak shifts slightly from 430 to 443 nm, likely due to the narrowed bandgap (Figs. S25, 4G, S8, and Discussion S6). In addition to the GSB and the photogenerated hole absorption signal (HA, ~ 470), two additional absorption peaks appear at BVO/FeOOH and BVO/FeOOH-GO_V_ film (Fig. 4H): one at ~ 480 nm, may attributed to the excited state absorption (ESA) signal [58, 59], and another at ~ 510 nm, attributed to surface trapped holes absorption [60]. Compared to BVO/FeOOH, the BVO/FeOOH-GO_V_ film shows a significant increase in signal intensity at 443, 470, 480, and ~ 510 nm, confirming a significantly reduced recombination dynamics [61].

The carrier dynamics of the samples were investigated by fitting the kinetics traces at ~ 443 nm (GSB), ~ 470 nm (HA) and ~ 510 nm (Trapped holes) (Figs. S26, 4I, and Tables S9-S11). The average charge carrier decay lifetime (*τ*_av_) at ~ 443 nm (GSB) extends from 628.44 ps for BVO to 824.48 ps for BVO/FeOOH and 1267.61 ps for BVO/FeOOH-GO_V_ (Fig. S26A, and Table S9). The increased charge carrier decay lifetime of the GSB signal indicates that GO_V_ can effectively reduce the band-to-band recombination. However, the charge carrier decay *τ*_av_ at ~ 470 nm (HA) is observed and calculated to be reduced from 4473.93 ps (BVO) to 3191.95 ps (BVO/FeOOH) and 2247.90 ps (BVO/FeOOH-GO_V_) (Fig. S26B, and Table S10), indicating the FeOOH-GO_V_ has the ability to quickly extract holes. These extracted holes are quickly transported to the FeOOH surface which will then be trapped by the abundant surface GO_V_; thus, it is meaningful to monitor the lifetime of trapped holes. The *τ*_av_ of trapped holes (~ 510 nm) is substantially extended to 9146.98 ps for BVO/FeOOH-GO_V_ compared to 5947.26 ps for BVO/FeOOH (Table S11), indicative of the long-lived photogenerated holes for water oxidation caused by surface GO_V_. Overall, these findings provide strong evidence that the photogenerated charge carrier dynamics can be greatly improved by loading FeOOH-GO_V_, thereby enhancing the PEC activity of BVO photoanode.

### Effect of GO_V_ on Electronic Structure of FeOOH OEC

**Fig. 5:**
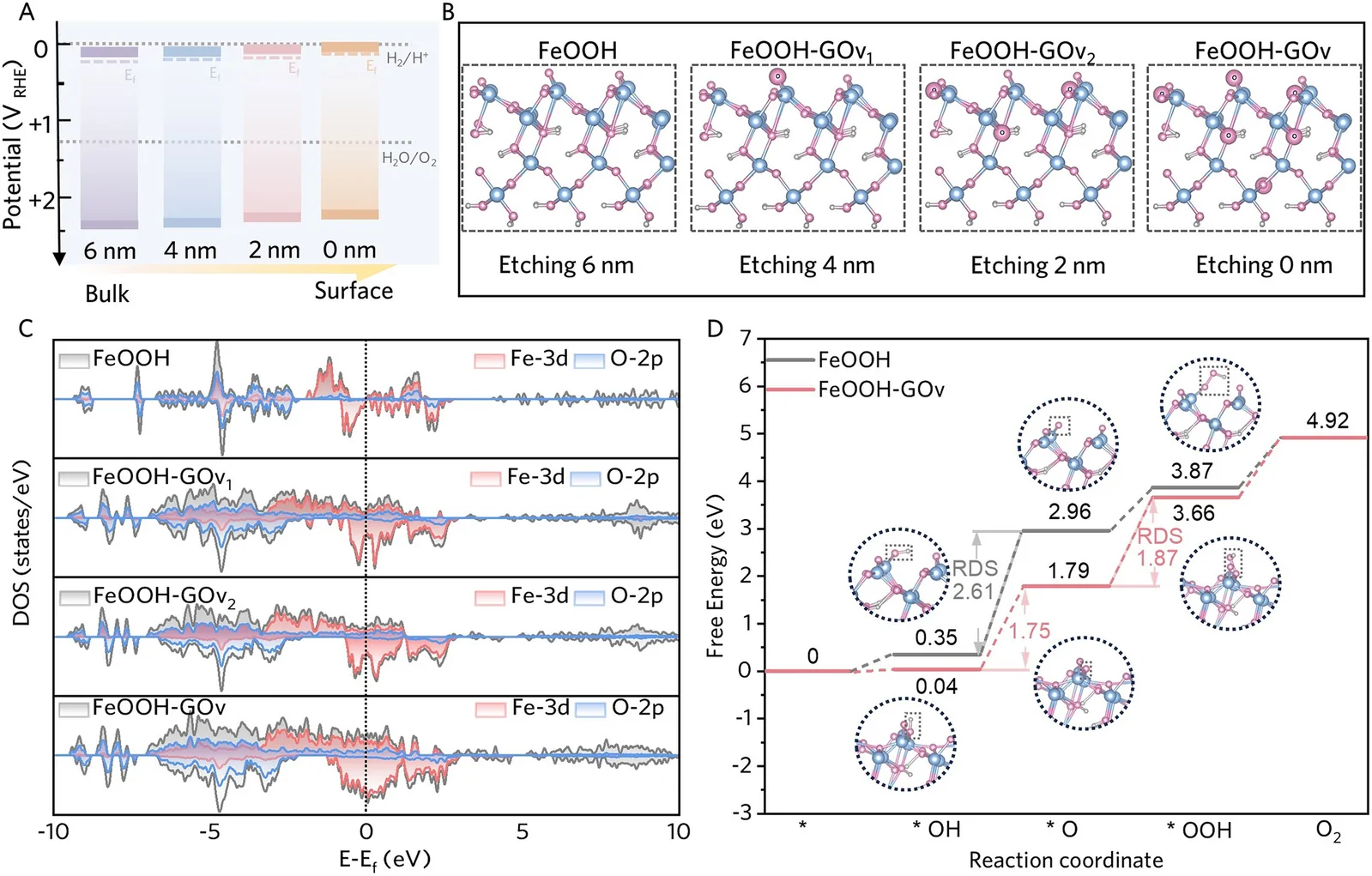
**A** Band structures of BVO/FeOOH-GO_V_ film with different etching depths. **B** Initial structural model of different FeOOH (blue balls: Fe atoms; pink balls: O atoms; gray balls: H atoms; the larger O atoms marked in the initial models were the deducted O atoms). **C** Calculated TDOS (gray), Fe-3*d* (red) and O 2*p* (blue) PDOS of different FeOOH, where the Fermi energy level is set to 0, **D** Calculated Gibbs free energy diagram of the OER process on the FeOOH and FeOOH-GO_V_ surface at U = 0 V

To explore the effect of GO_V_ on band structure of BVO-FeOOH films, the UPS measurements of BVO-FeOOH, BVO-FeOOH-GO_V_ films were carried out (Fig. S27). Based on the UPS (Figs. S12 and S27A–D) and UV–vis (Fig. S8) results, the band positions for BVO, BVO/FeOOH and BVO/FeOOH-GO_V_ are shown in Fig. S27E and Table S12. The incorporation of GO_V_ significantly alters the band structure by causing a significant upward shift in the valence band maximum (VBM), which is verified by the UPS measurements for BVO-FeOOH-GO_V_ film performed at multiple etching depths, as shown in Figs. 5A and S27C–E. The graphical representation visually confirms that GO_V_ creation progressively narrows the energy gap between the VBM and E_f_ (Fig. 5A), which signifies an increase in the thermodynamic driving force for hole transfer. The dynamic results observed by the above IMPS align with UPS-observed VBM upshifts and XPS-detected O_V_ concentration change, collectively indicating that GO_V_ creates the progressively upward-shifting valence band resulting a drift-assisted transport pathway, thus significantly improving the hole transport capability and increasing the charge injection efficiency.

The density functional theory (DFT) calculations were performed to further explore the specific electronic structure of FeOOH-GO_V_ with different O_V_ gradient distributions. The initial structural models are based on the distribution of O_V_ at four different etching depths, and the models at etching depths of 6, 4, 2, and 0 nm are labeled as FeOOH, FeOOH-GO_V1_, FeOOH-GO_V2_, and FeOOH-GO_V_, respectively (Fig. 5B, noting: the O_V_ concentration at 6 nm depth is close to that of FeOOH sample without PE treatment, therefore this model is considered to be FeOOH). The optimized structural models are present in Fig. S28. The calculated total density of states (TDOS) reveals that the VBM and the conduction band minimum (CBM) of different FeOOH are predominantly comprised of Fe-3*d* and O 2*p* orbitals, respectively (Fig. 5C). For the FeOOH-GO_V1_, FeOOH-GO_V2_, and FeOOH-GO_V_, the significant overlap of Fe-3*d* with the O 2*p* orbitals alters the VB electronic structure of FeOOH, leading to obvious upward shifts of the VBM of the FeOOH (Figs. 5C, S29, and Table S13). As the concentration of GO_V_ intensifies, the upward shift in VBM becomes increasingly pronounced, highlighting the critical role of GO_V_ in modulating the electronic structure for FeOOH. Besides, the comparative analyses of TDOS for four models reveal a pronounced enhancement in the DOS near the Fermi level specifically in FeOOH-GOv. Fe-3*d* in the FeOOH-GO_V_ exhibits a substantial contribution to the TDOS near the Fermi level, leading to a pronounced increase in the number of active electrons available for OER [62, 63]. Building on the aforementioned analyses, the OER process through different reaction pathway was simulated using DFT calculations. The calculated Gibbs free energy of FeOOH and FeOOH-GO_V_ for OER process are shown in Fig. 5D, where the transition from *OH to*O is the rate-determining step (RDS) in the OER process for FeOOH. The energy barrier for the transition from *OH to*O of FeOOH is determined to be 2.61 eV, while it decreases to 1.75 eV in FeOOH-GO_V_. The electronically and coordinatively unsaturated sites introduced by O_V_ facilitate H^+^ removal, thereby promoting the progression of *OH → *O step. However, the *O → *OOH step emerges as the new RDS, with a calculated Gibbs free energy barrier of 1.87 eV. The high concentration of O_V_ induces a strong electron-donating effect, which is sufficient to modify the adsorption behavior of intermediates and shift the RDS away from the *OH → *O step. The reduction in the RDS barrier from 2.61 to 1.87 eV provides strong theoretical evidence that the catalytic activity for OER is indeed improved on the FeOOH-GO_V_ surface, despite the shift in which specific step limits the rate. Hence, the OER catalytic activity of the MBVO/FeOOH films can be significantly enhanced.

Further precise Bader charge analysis for both the pristine FeOOH model and the FeOOH-GO_V_ models provides insight into the built-in electric field. Figure S30A depicts the FeOOH-GO_V_ structural model with the Bader charge values annotated for the Fe atoms nearest to the oxygen vacancies. The corresponding values for the pristine FeOOH model at identical positions are also indicated for direct comparison (Fig. S30B). In the FeOOH-GO_V_ model, the Bader charge values of most Fe atoms nearest to the oxygen vacancies are significantly lower than those in pristine FeOOH, confirming the vacancy-induced reduction of adjacent Fe atoms (Fe^3+^  → Fe^2+^). More importantly, owing to the gradient distribution of oxygen vacancies from the surface to the bulk, the average values of Bader charge for these Fe atoms exhibit a clearly increasing gradient (Table S14). The spatial variation rate of the charge difference is greater in the FeOOH-GO_V_ model, indicating more pronounced nanoscale charge separation and a steeper potential gradient. This enhanced charge gradient promotes a stronger built-in electric field directed from the bulk to the surface, which provides the fundamental driving force for the proposed “relay transport” mechanism, enhancing charge separation and supplying holes to surface reaction sites, thereby dramatically boosting the photocatalytic activity.

Based on the above calculation and experimental results, a mechanism is proposed to explain the improved PEC performance for BVO/FeOOH-GO_V_ photoanode. When BVO/FeOOH photoanode is illuminated, most of the photogenerated carriers are quickly transferred to the BVO surface. Simultaneously, these photogenerated holes will be transferred to the thick FeOOH layer. However, most of them are recombined before reaching the FeOOH surface for water oxidation reaction owing to the short hole diffusion length (Fig. S1B). When FeOOH is photoetched to generate abundant GO_V_, the hole transport capability is significantly enhanced, which is attributed to the progressive upward shift of the VBM within the spatial distribution by GO_V_ incorporation. This gradient band structure effectively creates a built-in electric field through valence band alignment engineering, which provides a directional driving force for hole transport from the BVO surface to the electrolyte interface. The field-driven transport mechanism reduces both the energy barrier for hole migration and the probability of charge recombination within the FeOOH layer, exhibiting characteristic “relay transport” behavior where holes are sequentially transferred across energy-matched states (Scheme 1B). This allows more holes to reach the FeOOH surface, contributing to OER reaction. Furthermore, the high-density surface O_V_ induced by GO_V_ incorporation creates abundant catalytic centers on the FeOOH surface, reducing the hindrance for the adsorption of H_2_O, thus boosting the OER activity of BVO/FeOOH-GO_V_ photoanode (Scheme 1B). In summary, the synergistic effects of enhanced hole transport kinetics through gradient band engineering and increased active site density collectively improve both the water oxidation activity and operational stability of the BVO/FeOOH-GO_V_ photoanode.

### Toward Generalized Application of GO_V_ Strategy: Requirements and Pathways

The broader application of the GO_V_ strategy relies on its generalizability, which remains limited due to strong dependence on carefully matched and coordinated optimization across the material system, synthesis protocol, characterization techniques, and target application. Specifically, the host material must have a crystal structure capable of sustaining high concentrations of oxygen vacancies without phase transformation or structural collapse. Ideal candidates are materials with multi-valent metal centers (e.g., Fe^3+^/Fe^2+^, V^5+^/V^4+^, Mo^6+^/Mo^5+^) that enable charge compensation during vacancy formation. Successful GO_V_ implementation also requires synthesis routes offering precise spatiotemporal control over reduction to create concentration gradients, rather than homogeneous distributions, often via post-treatment methods (e.g., controlled atmosphere calcination, chemical reduction) that regulate diffusion kinetics. Moreover, the strategy is most effective in systems where performance is limited by charge separation, as the GO_V_-induced built-in electric field directly promotes charge transport. Thus, beyond photoelectrochemical water splitting, this approach should be extended to related energy technologies such as photoelectrochemical CO_2_ reduction, where directed charge carrier movement is equally crucial.

## Conclusions

In conclusion, we have demonstrated a simple PE method for generating GO_V_ on FeOOH which is decorated onto BVO photoanodes. Experimental analyses confirm that the rich GO_V_ could enhance both the PEC activity and stability. More specifically, the existence of rich O_V_ can facilitate hole transport within FeOOH by the progressive upward shift of the VBM within the spatial distribution, and introduces numerous oxidation active sites on its surface by efficient hole trapping, thus effectively promoting the OER activities. Additionally, rapid hole extraction, enabled by the GO_V_, can effectively suppress photocorrosion by the instantaneous extraction of holes to participate in OER reaction. Benefiting from the surface GO_V_ of FeOOH, the BVO/FeOOH-GO_V_ photoanodes achieves an impressive photocurrent of 5.37 mA cm^2^ at 1.23 V_RHE_, along with excellent PEC stability (160 h). This work not only highlights the importance of the designing surface GO_V_ in the OECs to boost the OER activity and stability, but also provides design ideas for developing highly efficient PEC devices.

## Supplementary Information

Below is the link to the electronic supplementary material.

Supplementary file1 (DOCX 3726 KB)
