# Supplementary material for: FeOOH Cocatalysts with Gradient Oxygen Vacancy Distribution Enabling Efficient and Stable BiVO4 Photoanodes
Source: Nanomicro Lett. 2026 Jan 12;18:147. doi: 10.1007/s40820-025-01987-8 (PMC12791085; doi:10.1007/s40820-025-01987-8)
Supplement: Supplementary file 1 — Supplementary file1 (DOCX 3726 KB) [file 40820_2025_1987_MOESM1_ESM.docx]

Supporting Information for

**FeOOH Cocatalysts with Gradient Oxygen Vacancy Distribution Enabling Efficient and Stable BiVO_4_ Photoanodes**

Shiyuan Wang^1^, Mengjia Jiao^1^, Qian Ye^1^, Jie Jian^1^*, Fan li^2^, Guirong Su^3^, Lu Zhang^4^, Ziying Zhang^5^, Zelin Ma^1^, Jiulong Wang^1^, Yazhou Shuang^1^, Fang Wang^1^, Yalong Song^1^, Lichao Jia^4^ and Hongqiang Wang^1^*

^1^ State Key Laboratory of Solidification Processing, Center for Nano Energy Materials, School of Materials Science and Engineering, Northwestern Polytechnical University and Shaanxi Joint Laboratory of Graphene (NPU), Shaanxi Laboratory for Advanced Materials, Xi'an 710072, P. R. China

^2^ School of Physics and Information Technology, Shaanxi Normal University, Xi'an 710119, P. R. China

^3^ College of Materials Science and Engineering, Hohai University, Changzhou 213200, P. R. China

^4^ School of Materials Science and Engineering, Shaanxi Normal University, Xi’an 710119, P. R. China

^5^ International Research Center for Renewable Energy, Xi’an Jiaotong University, Xi'an 710049, P. R. China

*Corresponding authors. E-mail: [jianjie112@nwpu.edu.cn](mailto:jianjie112@nwpu.edu.cn) (Jie Jian); [hongqiang.wang@nwpu.edu.cn](mailto:hongqiang.wang@nwpu.edu.cn) (Hongqiang Wang)

**S1 Experimental Section**

**Materials.** Dimethyl sulfoxide (DMSO) was obtained from Sigma-Aldrich. Bismuth nitrate pentahydrate (Bi(NO_3_)_3_·5H_2_O, >98%), Sodium sulfite (Na_2_SO_3_, >97%), p-benzoquinone (C_6_H_4_O_2_, >99%) and Potassium hydroxide (KOH, >85%) were obtained from Sinopharm Chemical Reagent Co., Ltd.. Iron chloride hexahydrate (FeCl_3_·6H_2_O, >99%) was purchased from Macklin Inc.. Vanadium acetylacetone oxygen (VO(acac)_2_, >99%) was purchased from Acros Organics and Fisher Scientific. Fluorine-doped tin oxide (FTO) substrates (~ 30 Ω) was obtained from Pilkington. Deionized water with a resistance of 18.2 MΩ·cm was produced by a Water Purification System (Merck Millipore, Direct-Q5 UV). All materials were used as received.

**Preparation of the BVO films.** The BiVO_4_ (BVO) photoanodes were fabricated by the electrochemical deposition reported elsewhere^1^. Briefly, 46 mM p-benzoquinone (C_6_H_4_O_2_) was dissolved in ethanol and sonicated for 10 minutes to obtain a yellow green transparent clear solution, denoted as solution A. 20 mM Bi(NO_3_)_3_·5H_2_O and 400 mM KI were dissolved in DI water, followed by adding 38 mM nitric acid to adjust the pH to 4.7, and the obtained orange transparent clear solution was denoted as solution B. Then, solution A was slowly added to solution B to obtain a bright red transparent and clear solution. Next, a three electrodes deposition system was used for electrochemical deposition. The working electrode is FTO glass treated by UV for 15 min, the counter electrode is Pt sheet, and the reference electrode is Ag/AgCl. The voltage was set to -0.1 V and the deposition time is controlled to be 600 s to obtain BiOI film. Then, 0.2 M VO(acac)_2_ was dissolved in DMSO and sonicated for 5 minutes to fully dissolve. The 250 µL of above solution was dripped onto the surface of the BiOI film, and subsequently putted on a hot bench of 120 °C for 15 minutes. Then, the obtained thin film was annealed at 450 °C for 2 hours with a heating rate of 2 °C /min. After natural cooling, the film was then soaked in a 1.0 M NaOH solution for 20 minutes to remove excess V_2_O_5_ on the surface. Finally, the film was rinsed with DI water and dried with N_2_.

**Preparation of the BVO/FeOOH film.** The BVO photoanode was placed in a 10 ml 0.01 M FeCl_3_ H_2_O-dimethyl sulfoxide (DMSO) mixed solution with the H_2_O-DMSO ratios stetted as 3/1 for 10 h at 50 °C to obtain BVO/FeOOH with different phase film. Then, the films were washed with DI water and dried with nitrogen.

**Preparation of the BVO/FeOOH-GO_V_ film.** The as prepared BVO/FeOOH photoanodes were immersed in the 1M KPi buffer solution with 0.1M Na_2_SO_3_ for different times under illumination by A Xe 500 W lamp (AM 1.5G).

**Material Characterization.** The morphologies of all films were evaluated through a field emission scanning electron microscopy (FESEM, FEI Nova Nano SEM 450). X-ray diffraction (XRD) spectra were used to characterize the crystal structure of all films by a D8 ADVANCE (Bruker). The Raman spectra of all films were identified using a Renishaw in Via Raman microscope with a 532 nm laser. Ultraviolet–visible (UV–vis) spectra of all films were performed to measure the absorbance of all samples by a Perkin-Elmer Lambda 35 UV-vis-NIR spectrophotometer. Transmission electron microscopy (TEM) and high-resolution transmission electron microscopy (HRTEM) were performed through a FEI Talos F200X microscope. The FTIR spectra (4000 to 500 cm-1) were recorded on a Jasco FT/IR-6100 FTIR. Scanning transmission electron microscopy coupled with electron energy-loss spectroscopy (STEM-EELS) were recorded on a FEI Themis Z. X-ray photoelectron spectroscopy (XPS) was recorded on a ULVAC-PHI (PHI 5000 VersaProbe III) XPS system, the depth of each sputtering process was controlled by time (etch rate ~ 0.1 nm/s), and all peak were calibrated by the C 1 s peak (284.8 eV) as a reference. The photoluminescence (PL) spectra were carried out using a PL spectrometer (FLS 980, Edinburgh) with excitation laser of 310 nm. (TAS) measurements of all films were performed with a commercial TA system (Time-Tech Spectra, LLC). Electron paramagnetic resonance (EPR) measurements were conducted on Bruker EMXPLUS operating at room temperature. The intensity modulated photocurrent spectroscopy (IMPS) of all films was recorded on a Modulab Solartron Analytical potentiostat, model 2200 A. A cold white LED that drove illumination of 455 nm was adopted as light source in 1.0 M potassium phosphate buffer solution at 1.23 V_RHE_. The light intensity was adjusted to 68.93 mW cm^-2^, and it was modulated by 10% in the range of 100 kHz to 0.1 Hz.

**Photoelectrochemical characterization.** The photoelectrochemical measurements were carried out at room temperature by a three-electrode test system and an electrochemical workstation (CHI660E). In the three-electrode test system, all the above MBVO photoanodes in this work were used as working electrodes, and the exposed irradiation area for PEC measurements was fixed as 0.25 cm^2^, while Ag/AgCl electrode and Pt electrode were used as reference and counter electrodes, respectively. 1.0 M potassium phosphate buffer with and without 0.1 M Na_2_SO_3_ aqueous solution (pH=7) were adopted as the electrolyte, respectively. A Xe 500 W lamp (CEL-S500, CEAULIGHT) adjusted to 100 mW cm^-2^ via an AM 1.5G filter was used as the light source. Photocurrent vs voltage (I-V) curves were recorded by scanning rate of 10 mV s^−1^ with the potential range from -0.6 to 0.8 V vs. Ag/AgCl. The cyclic voltammetry (CV) measurements were carried out at 1.23 V_RHE_, and the scan rate was 0.02 to 0.1 mV s^-1^. Electrochemical impedance spectroscopy (EIS) spectra were collected by frequency from 10 Hz to 10^6^ Hz at 1.23 V_RHE_. Mott-Schottky (MS) spectra were characterized with the potential range from -0.6 to 0.6 V vs. Ag/AgCl. The incident-photon-to-current conversion efficiency (IPCE) was measured using the same three-electrode cell described above at 1.23 V_RHE_ under monochromatic irradiation from a Xe lamp equipped with bandpass filters. Open circuit potentials (OCP) of the photoanodes were measured in 1.0 M potassium phosphate buffer (pH=7).

**Calculations.** All reversible hydrogen electrode (RHE) in this work were converted using the Nernst equation^1^:

| V_RHE_ = V_Ag/AgCl_ + 0.0591×pH + 0.197 (V) | (S1) |
| --- | --- |

where V_RHE_ refers to the potential versus RHE. V_Ag/AgCl_ is the potential versus Ag/AgCl.

IPCE, the absorbed photon-to-current conversion efficiency (APCE) and the applied bias photon-to-current efficiency (ABPE) values can be calculated using the following equations, resepectivly^2^:

| IPCE (%) = (J×1240) / (λ×P_light_)×100% | (S2) |
| --- | --- |
| APCE (%) = IPCE (%) / LHE×100% | (S3) |
| ABPE (%) = J×(1.23-V_app_)/P_light_ ×100% | (S4) |

where J is the photocurrent density of MBVO films (mA cm^-2^), λ is the incident light wavelength (nm), and P_light_ represents the power density of incident light. V_app_ is the applied external potential.

The carrier density (Nd) can be calculated according to the MS spectra by the following equation^3^:

| Nd = (2/eε_0_ε)×(d(1/C^2^) / dVs)^-1^ | (S5) |
| --- | --- |

where e, ε_0_, ε, C and Vs is the electronic charge (1.602 × 10^−19^ C), vacuum permittivity (8.854 × 10^−12^ F m^−1^), relative permittivity (68 F m^-1^ for BiVO_4_), the space charge capacitance (F cm^−2^, obtained from MS curves) and the applied potential (V), respectively^4^.

| η_inj_ =J (H_2_O) / J (Na_2_SO_3_) | (S6) |
| --- | --- |

where J (H_2_O) is the photocurrent density measured in 1M potassium phosphate buffer and J (Na_2_SO_3_) is the photocurrent density measured in 1M potassium phosphate buffer with 0.1M Na_2_SO_3_ (pH = 7).

The τ_n_ is calculated by the equation as follows:

| τ_n_ = -K_B_T /e(dOCP/dt) | (S7) |
| --- | --- |

Where the τ_n_, K_B_, T, e, and dOCP/dt are the carrier transfer lifetime, Boltzmann's constant, temperature, charge of single electron, and derivative of the OCP transient decay, respectively.

The charge transport time (τ_d_) can be gained from using the following equation^5^:

| τ_d_ =(2π*f*_IMPS_)^-1^ | (S8) |
| --- | --- |

Where *f*_IMPS_ is the frequency at the minimum imaginary component in the IMPS spectra.

According to the low frequency intercept of the IMPS plots, the represents k_trans_/(k_trans_+k_rec_), the charge transfer efficiencies (K_trans_/(K_rec_ + K_trans_)) can be obtained.

The (K*_tran_* + K*_rec_*) can be calculated using the following equation^5^:

| (2π*f*_max_)^-1^ = K_tran_ + K_rec_ | (S9) |
| --- | --- |

**Theoretical calculations.** Density functional theory (DFT) as implemented in the Vienna Ab-initio Simulation Package (VASP) ^6^ was employed to the optimize geometry structures. The (010) surface of FeOOH (Orthorhombic, Pbnm No. 62) was modelled with 15 Å vacuum. The supercell had a volume of 3035.917380 Å³, with lattice parameters: a = 9.0534 Å, b = 13.7937 Å, and c = 24.3107 Å. The projected augmented wave (PAW) potentials were used to describe the ionic cores and account for the valence electrons using a plane wave basis set with a kinetic energy cutoff of 450 eV. The electronic energy was considered self-consistent when the energy change was smaller than 10^-5^ eV. Geometry optimization was considered convergent when the force change was smaller than 0.04 eV/Å. The van der Waals interactions were considered using the method of the Grimme (DFT+D3) ^7^. The Brillouin-zone integration was sampled with a Monkhorst-Pack mesh of 2×2×1 during the structural relaxation calculations.

The Gibbs free energy of an adsorbate on the surface was calculated using the following equation:

| G = E + ZPE − TS | (S10) |
| --- | --- |

where E is the total energy, ZPE is the zero-point energy, T is 298.15 K, and S is the entropy.

The OER process was modeled as follows:

| *+ H_2_O→ OH* + H^+^ +e^–^ | (S11) |
| --- | --- |
| OH*→ O* + H^+^ + e^–^ | (S12) |
| O* +H_2_O → OOH* + H^+^ + e^–^ | (S13) |
| OOH* → O_2_ + H^+^ + e^–^ | (S14) |

where the * represents the adsorption sites of the surface, and OH*, O*, and OOH* represent the corresponding adsorption intermediates, respectively.

**S2** **Supplementary Figures and Tables**

**
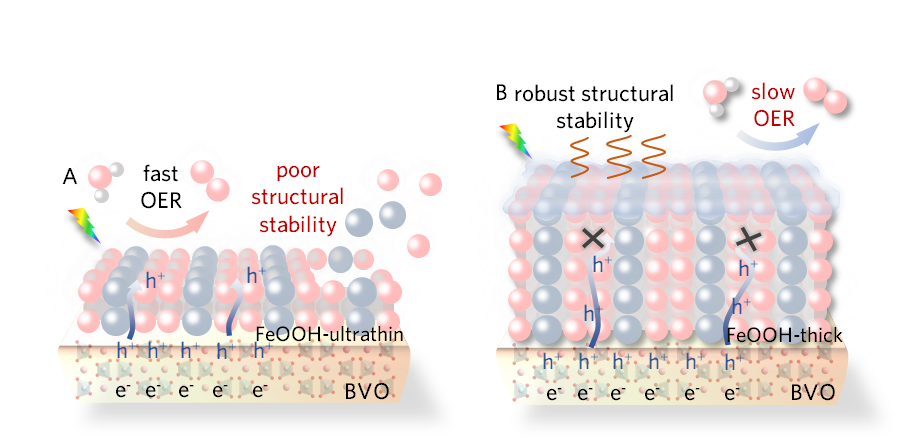
**

**Fig. S1** Illustration of (**A**) the fast OER and poor structural stability of ultrathin FeOOH layer and (**B**) the slow OER and robust structural stability of thick FeOOH layer decorated on BVO photoanodes


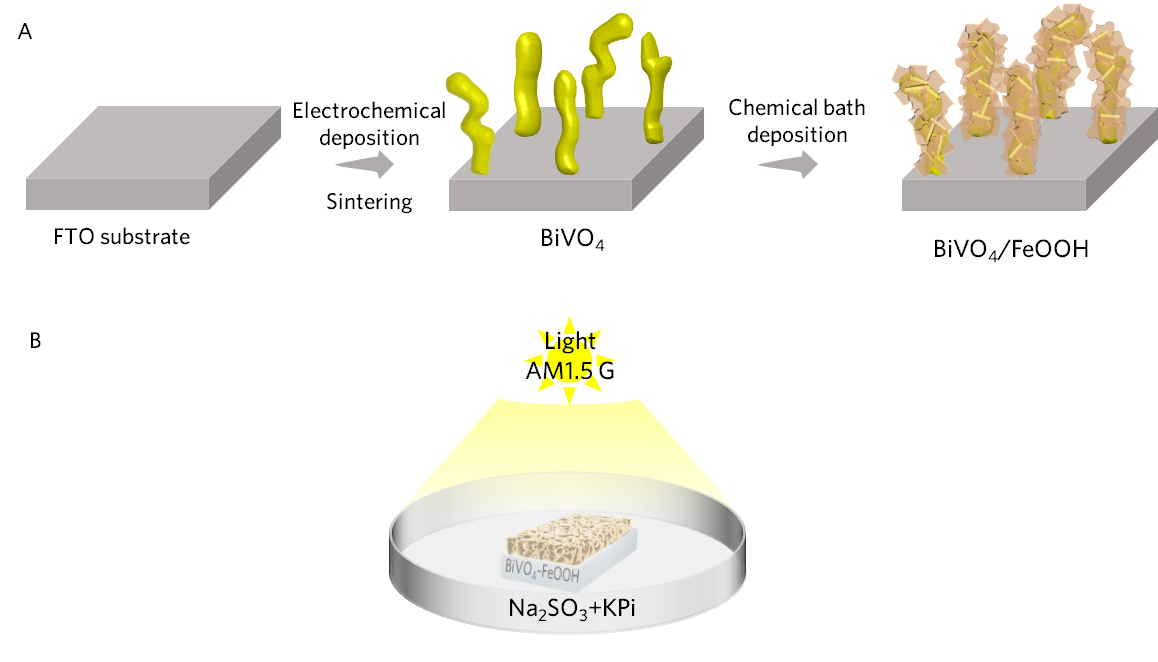


**Fig. S2** (**A**) The fabrication process of BVO/FeOOH films, (**B**) PE process for BVO/FeOOH film


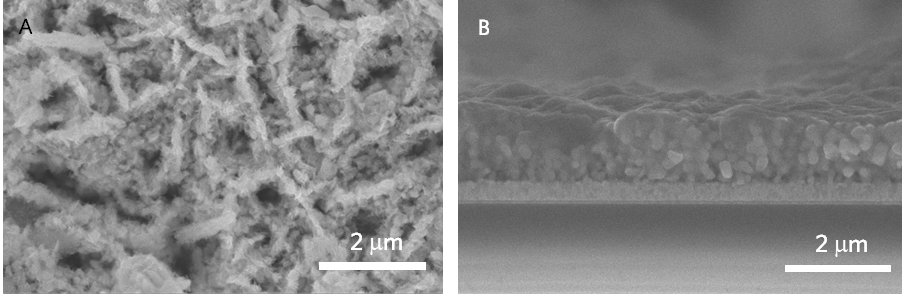


**Fig. S3** (**A**) SEM image and (**B**) cross cross-section SEM image of BVO/FeOOH photoanodes


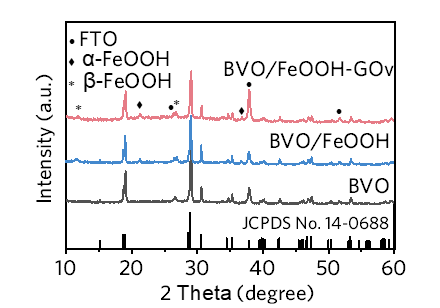


**Fig.** **S4** XRD patterns of BVO, BVO/FeOOH and BVO/FeOOH-GO_V_ films


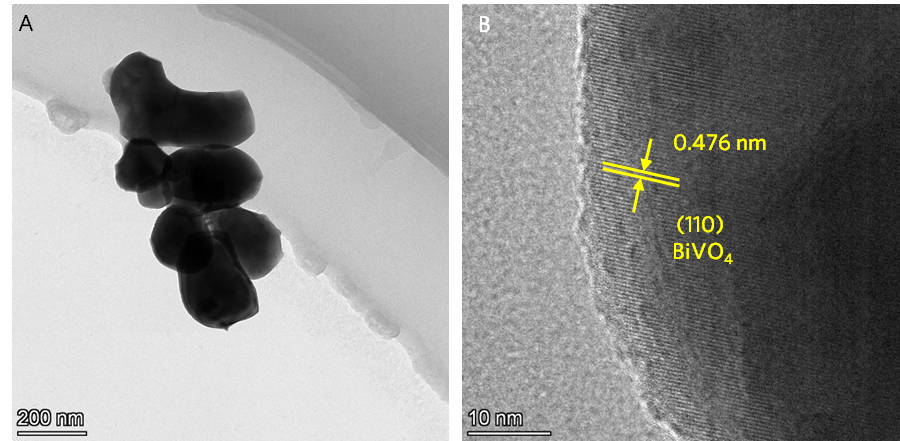


**Fig. S5** (**A**) TEM image and (**B**) HRTEM image of BVO film


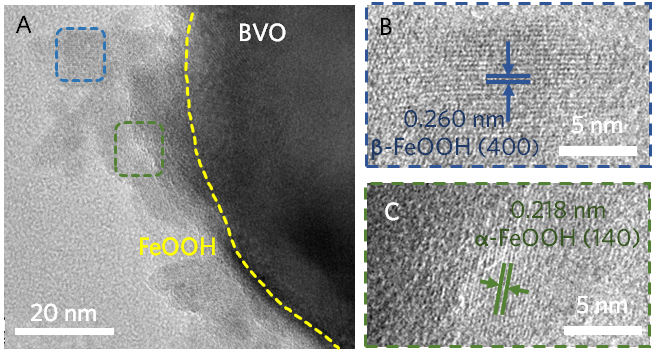


**Fig.** **S6** (**A**) HRTEM image of BVO/FeOOH photoanode. (**B**) HRTEM image of area labeled in bule in (**A**). (**C**) HRTEM image of area labeled in green in (**A**)


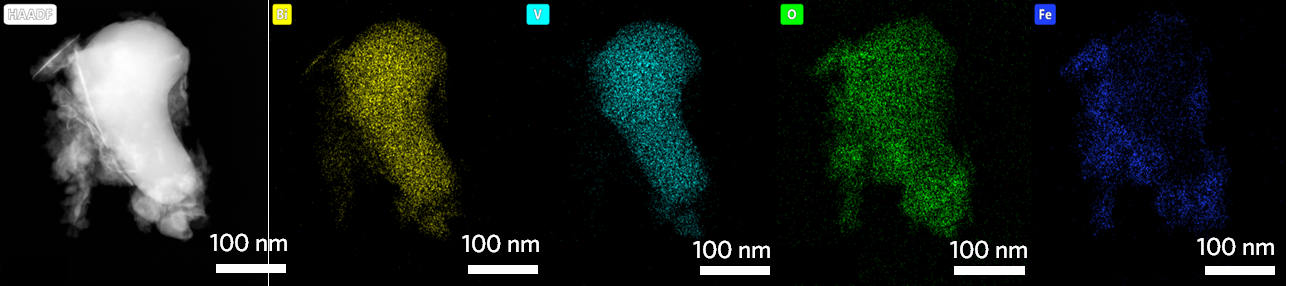


**Fig.** **S7** TEM-EDS analysis of BVO/FeOOH-GO_V_ film


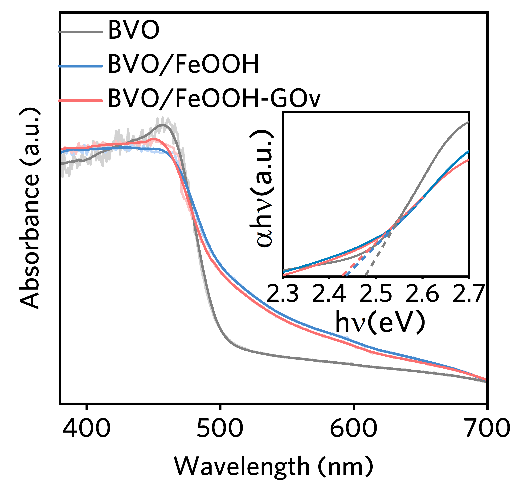


**Fig.** **S8** UV-vis absorption spectra of the bare BVO, BVO/FeOOH and BVO/FeOOH-GO_V_ films (insert: Tauc plots derived from UV-vis absorption spectra)


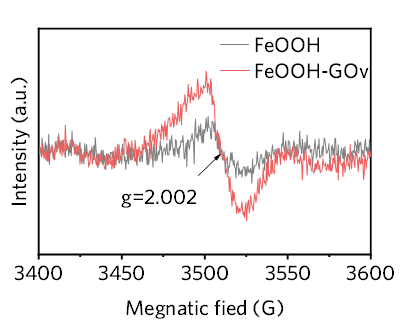


**Fig.** **S9** EPR spectra of FeOOH and FeOOH-GO_V_


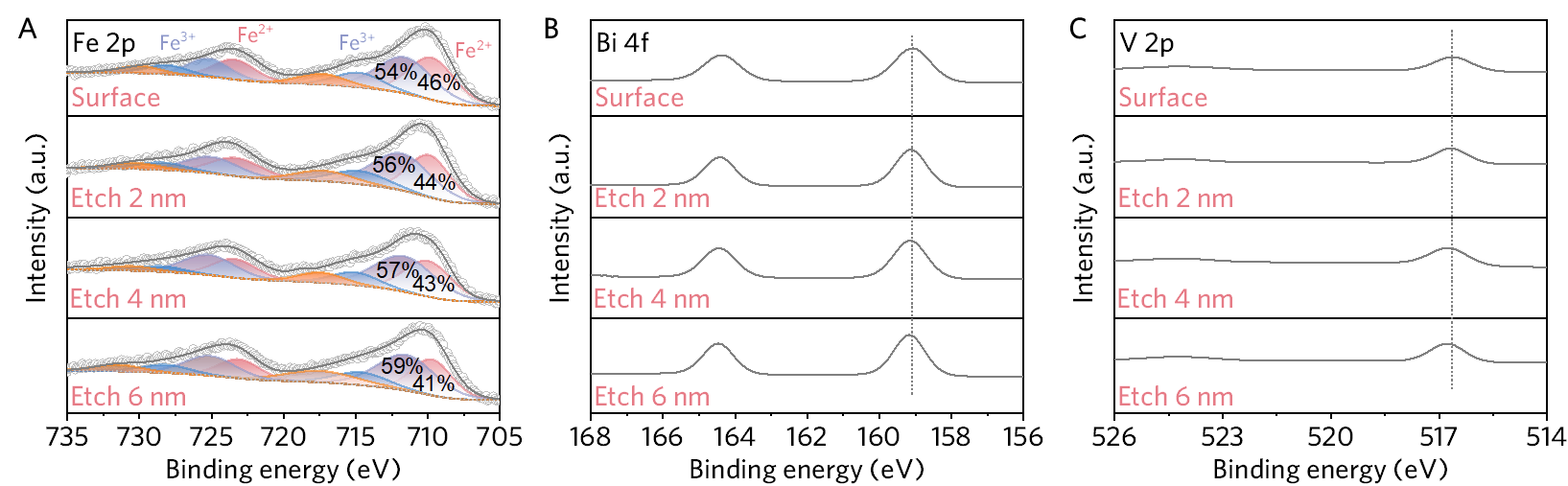


**Fig.** **S10** Depth profiles of (**A**) Fe 2p, (**B**) Bi 4f and (**C**) V 2p for BVO/FeOOH-GO_V_ film


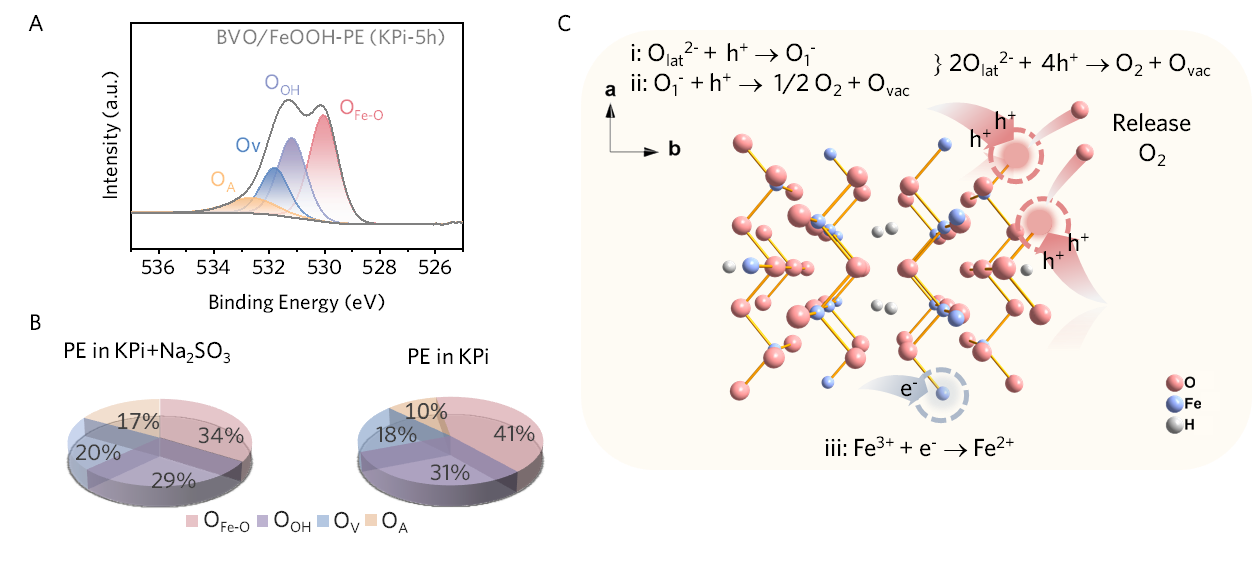


**Fig.** **S11** (**A**) XPS of O 1s of BVO/FeOOH film photoetched in KPi without 0.1M Na_2_SO_3_. (**B**) The Pie chart of O_L_, O_V_ and O_A_ derived from the O 1s of BVO/FeOOH-GO_V_ film photoetched in KPi with and without 0.1M Na_2_SO_3_. (**C**) The illustration of the generation of O_V_ in FeOOH during the photoetch treatment process in KPi

**Discussion S1:** The XPS quantification reveals that there is a large amount of Ov in BVO/FeOOH-PE (KPi+SO_3_^2-^) photoanode, while less O_V_ in BVO/FeOOH-PE (KPi) photoanode. This disparity fundamentally excludes that the presence of O_V_ is due to the Fe³⁺ reduction (Fe³⁺ → Fe²⁺), as such a Fe^3+^ reduction-mediated process would yield a comparable O_V_ concentration in BVO/FeOOH photoanodes regardless of sulfite presence. The similar photoreduction has been reported for other semiconductors [S8, S9]. Their results reveal that the holes can actively oxidize O_lat_^2-^ and release O_2_, which results in the generation of O_V_.


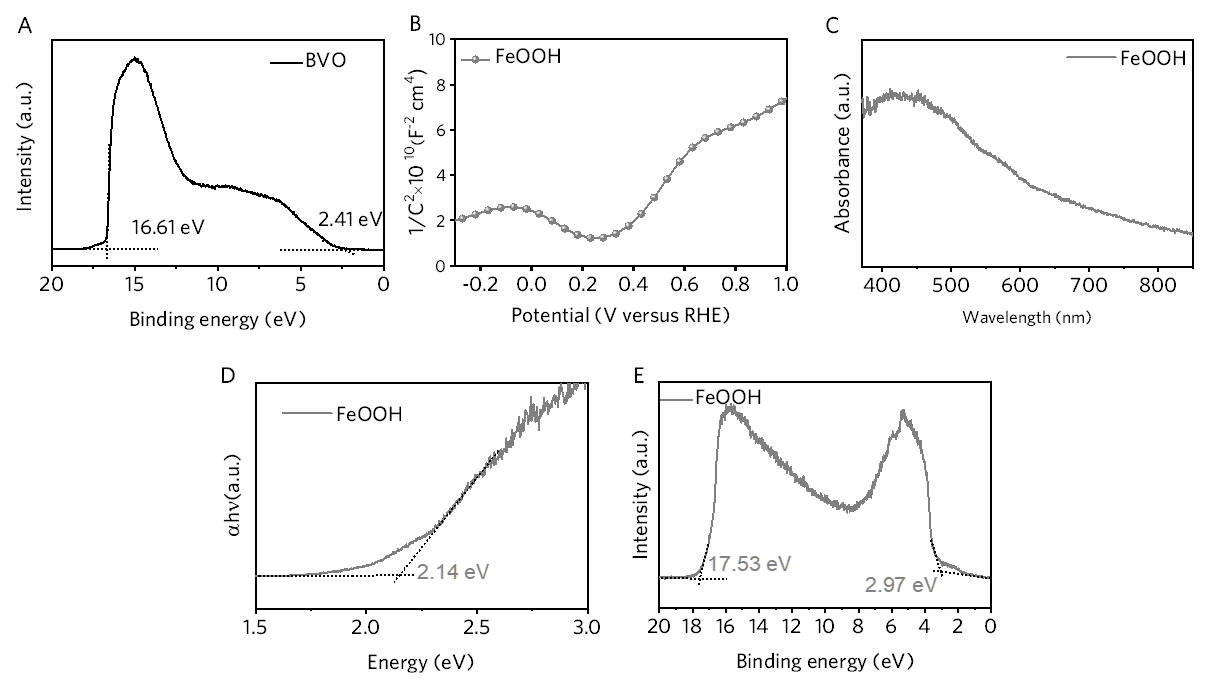


**Fig.** **S12** (**A**) UPS of BVO photoanode, (**B**) M-S plot, (**C**) UV-vis absorption spectroscopy, (**D**) Tauc plots derived from UV-vis absorption spectroscopy, (**E**) UPS of FeOOH


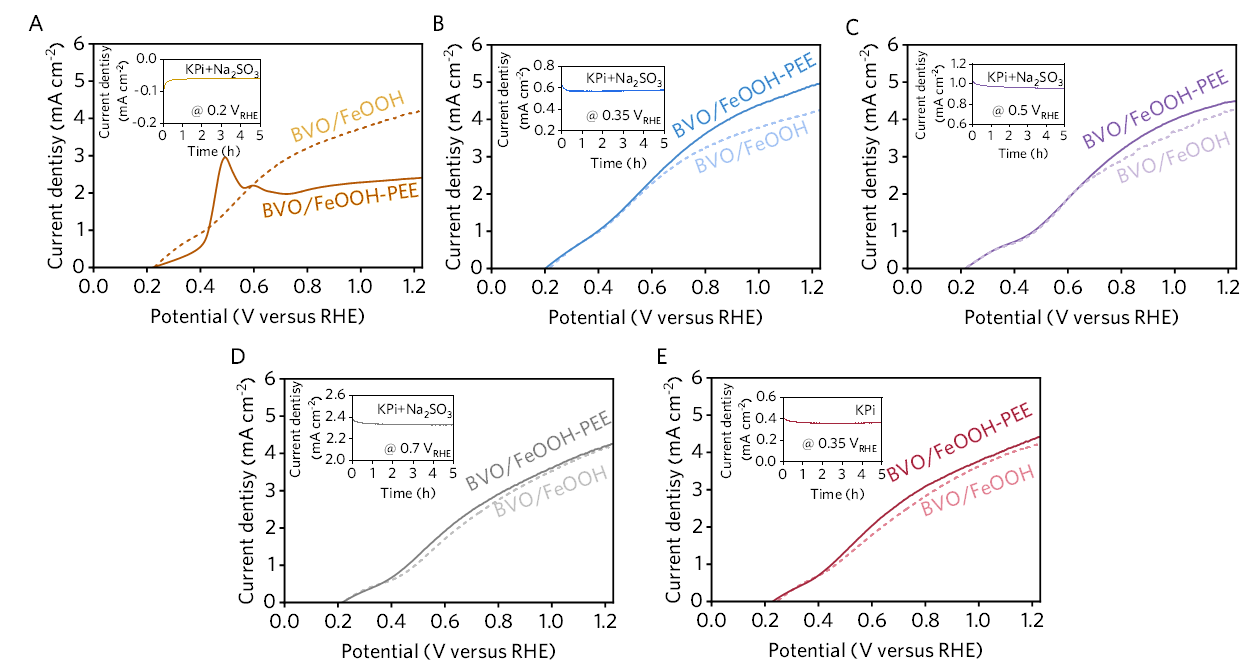


**Fig.** **S13** J-V curves for BVO/FeOOH films before (dash line) and after (solid line) photoelectric etching (PEE) treatment at (**A**) 0.2 V_RHE_ in 1 M KPi buffer with 0.1M Na_2_SO_3_, (**B**) 0.35 V_RHE_ in 1 M KPi buffer with 0.1M Na_2_SO_3_, (**C**) 0.5 V_RHE_ in 1 M KPi buffer with 0.1M Na_2_SO_3_, (**D**) 0.7 V_RHE_ in 1 M KPi buffer with 0.1M Na_2_SO_3_ and (**E**) 0.35 V_RHE_ in 1 M KPi buffer under AM 1.5G illumination (each insertion graph is the I-T curve corresponding to the potentiostatic PEE).

**Discussion S2:** Based on the flat band potential of the BVO/FeOOH photoanode, we selected four applied biases: 0.2 V_RHE_, 0.35 V_RHE_, 0.5 V_RHE_ and 0.7 V_RHE_, respectively. At 0.2 V_RHE_ (below the flat band potential), BVO/FeOOH photoanode exhibits a reduction current. After 5 hours, this condition leads to BVO/FeOOH photoanode failure due to cathodic corrosion (**Fig.** S11A). Therefore, the applied bias was set to be higher than the flat band potential, while remaining sufficiently low to avoid significant electrochemical oxidation or dissolution of BVO or FeOOH.


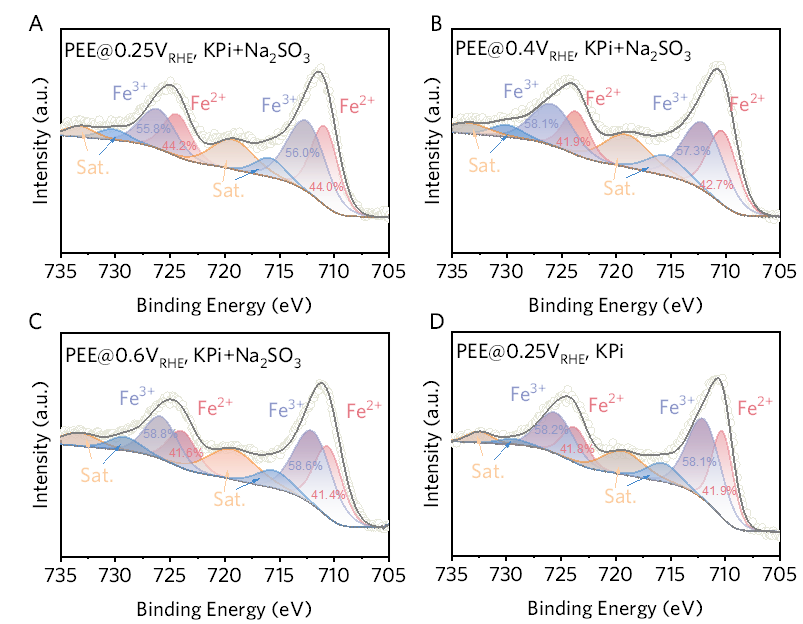


**Fig.** **S14** XPS of Fe 2p peaks of photoelectric etched BVO/FeOOH at (**A**) 0.35 V_RHE_ in 1.0 M KPi buffer with 0.1M Na_2_SO_3_, (**B**) 0.5 V_RHE_ in 1 M KPi buffer with 0.1M Na_2_SO_3_, (**C**) 0.7 V_RHE_ in 1 M KPi buffer with 0.1M Na_2_SO_3_ and (**D**) 0.35 V_RHE_ in 1 M KPi buffer under AM 1.5G illumination


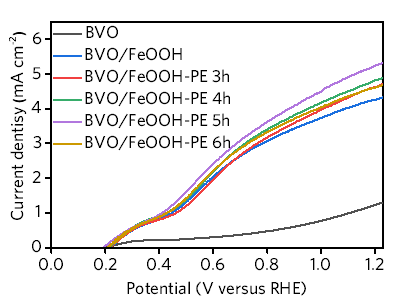


**Fig. S15** J-V curves for BVO/FeOOH films photoetched with different time


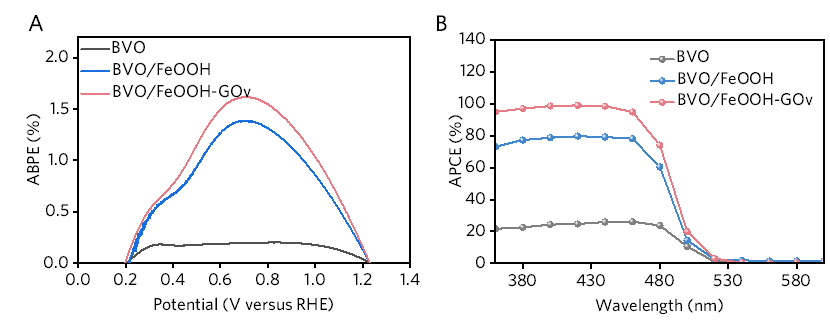


**Fig.** **S16** (**A**) ABPE and (**B**) APCE for BVO, BVO/FeOOH and BVO/FeOOH-GO_V_ films


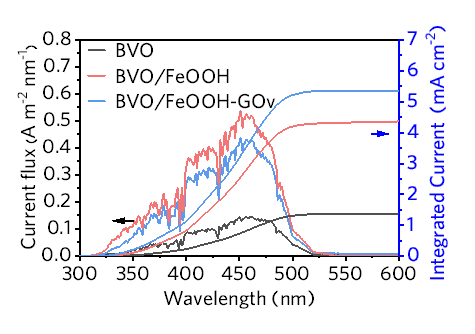


**Fig.** **S17** The current flux by integrating IPCE over the photon flux of AM 1.5G and the corresponding integrated current density of BVO, BVO/FeOOH and BVO/FeOOH-GO_V_ photoelectrodes at the potential of 1.23 V_RHE_


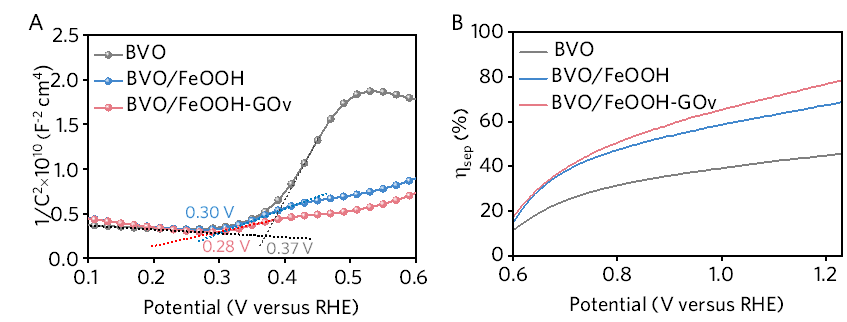


**Fig.** **S18** (**A**) M-S plots and (**B**) Charge separation efficiencies of BVO, BVO/FeOOH and BVO/FeOOH-GO_V_ films


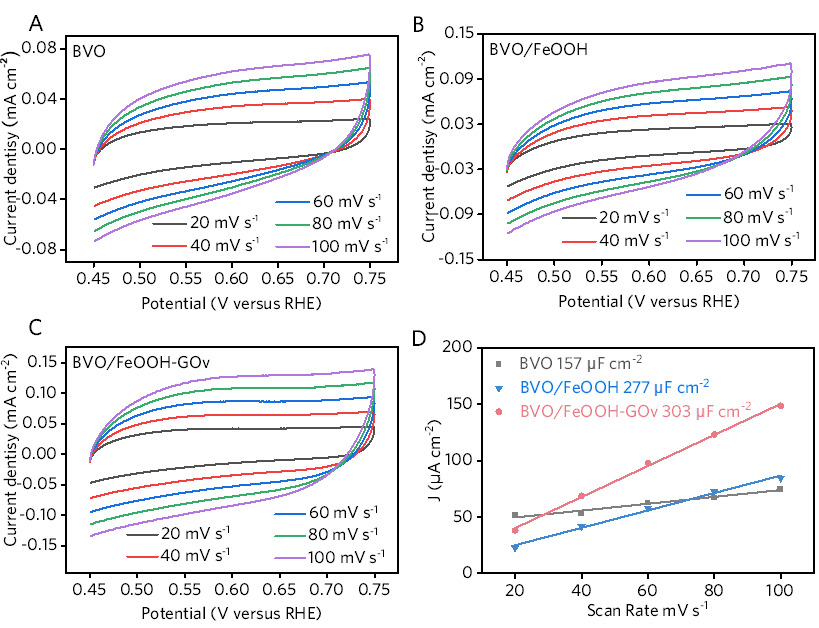


**Fig.** **S19** Cyclic voltammetry curves of (**A**) BVO, (**B**) BVO/FeOOH and (**C**) BVO/FeOOH-GO_V_ films. (**D**) the Δcurrent density-scan rate curves of BVO, BVO/FeOOH and BVO/FeOOH-GO_V_ films


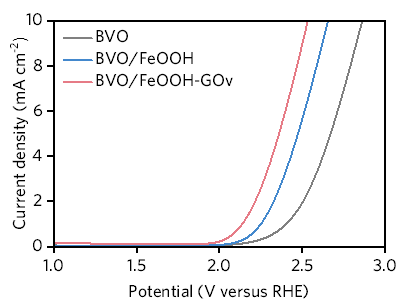


**Fig.** **S20** J-V curves of BVO, BVO/FeOOH and BVO/FeOOH-GO_V_ films in dark


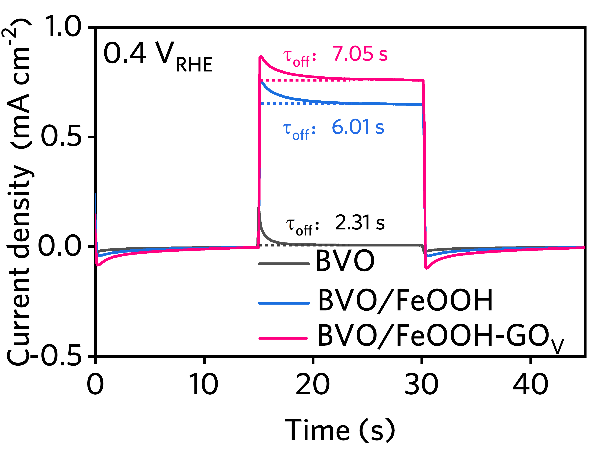


**Fig.** **S21** Chronoamperometry curve of BVO, BVO/FeOOH and BVO/FeOOH-GO_V_ films under chopped illumination

**Discussion S3:** Defect states predominantly govern charge carrier recombination kinetics and transport dynamics, with their most pronounced influence manifested in the photocurrent decay profile following illumination cessation (turn-off transient). BVO exhibits rapid photocurrent decay (~2.31 s) post-illumination cessation, characteristic of defect states acting as recombination centers. FeOOH deposition markedly slows decay kinetics (~6.01 s), confirming suppressed surface recombination and optimized interfacial charge transfer between BVO and FeOOH. BVO/FeOOH-GO_V_ demonstrates the slowest decay (~7.05 s, a little longer than BVO/FeOOH), exceeding both BVO and BVO/FeOOH, attributable to its GO_V_ architecture. This engineered GOv distribution enhances charge extraction while avoiding deep-level defects within FeOOH that form under random/high-concentration O_V_ conditions.


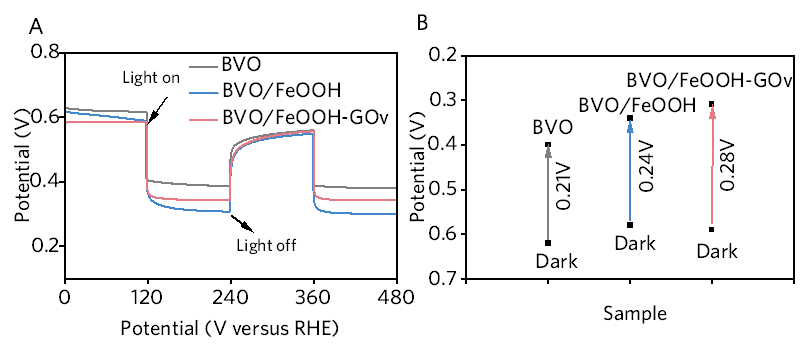


**Fig.** **S22** (**A**) Photovoltage response and (**B**) photovoltage of BVO, BVO/FeOOH and BVO/FeOOH-GO_V_ films


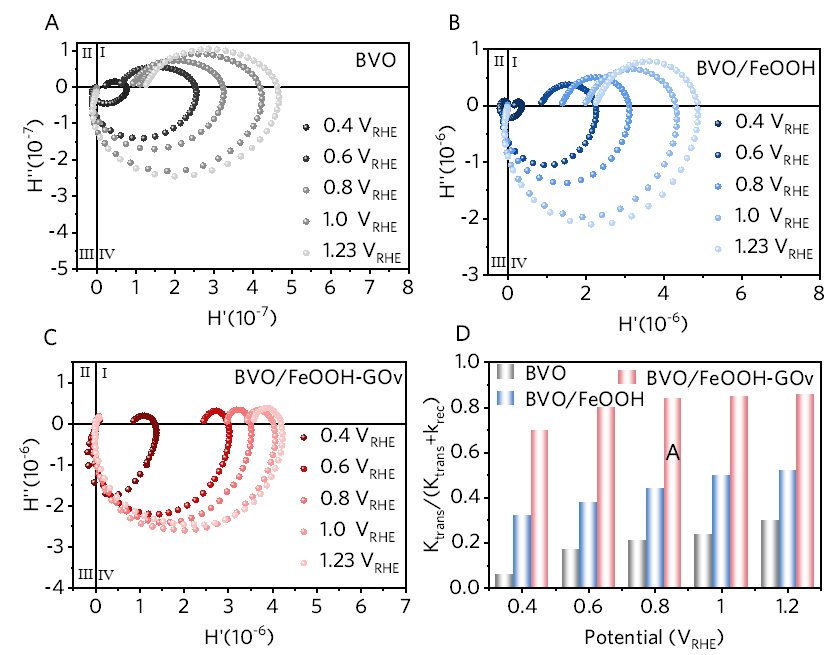


**Fig. S23** IMPS plots of (**A**) BVO, (**B**) BVO/FeOOH and (**C**) BVO/FeOOH-GO_V_ films measured at applied bias potentials ranging from 0.4 V_RHE_ to 1.23 V_RHE_, (**D**) the charge transfer efficiencies (K_trans_/(K_rec_ + K_trans_)) derived from the IMPS plots of BVO, BVO/FeOOH and BVO/FeOOH-GO_V_ films

**Discussion S4** The high k_rec_​ value of BVO photoanode shown in **Fig.** 3F at low bias potential can be attributed to three main factors. First, the weaker built-in electric field at a lower bias potential may lead to vigorous recombination of photogenerated electrons and holes in the BVO bulk. Second, the unsaturated surface states of the BVO films can trap charge carriers at low bias potentials, significantly increasing surface recombination. Third, the slower water oxidation reaction rate at low bias potentials can result in the accumulation of photogenerated holes, further exacerbating recombination. However, as the applied bias potential increases, the enhanced electric field strength, saturation of surface states, and accelerated reaction kinetics collectively suppress recombination, leading to a decreased and almost constant recombination constant.


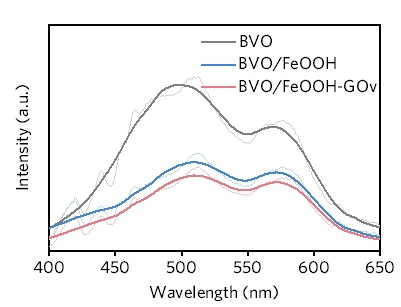


**Fig.** **S24** PL spectra of BVO, BVO/FeOOH and BVO/FeOOH-GO_V_ films

**Discussion S5:** There are two peaks could be clearly identified. The peak at 500 nm is assigned to inter-band radiative recombination of holes of BVO^10^, and the peak at 570 nm is attributed to the near-band-edge emission of BVO^11^. The strong PL peak of BVO indicates a relatively high electron-hole recombination ratio. Evidently, the PL peak intensities of BVO/FeOOH-GO_V_ film have been reduced, providing strong evidence that the FeOOH-GO_V_ inhibits carrier recombination of BVO surface most effectively.


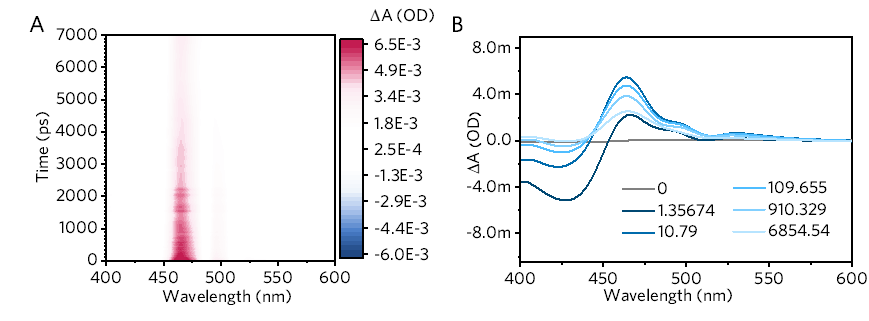


**Fig. S25** (**A**) TAS spectra and (**B**) TAS at selected delay times of BVO film

**Discussion S6:** The bare BVO principally presents two characteristic bands, a ground state bleaching (~430 nm, GSB) peak, attributed to the depopulation of the ground state due to band gap transition, which is correlated to the VB free hole dynamics, and an absorption peak (~470 nm, HA) corresponding to the absorption of photogenerated holes [S12, S13].


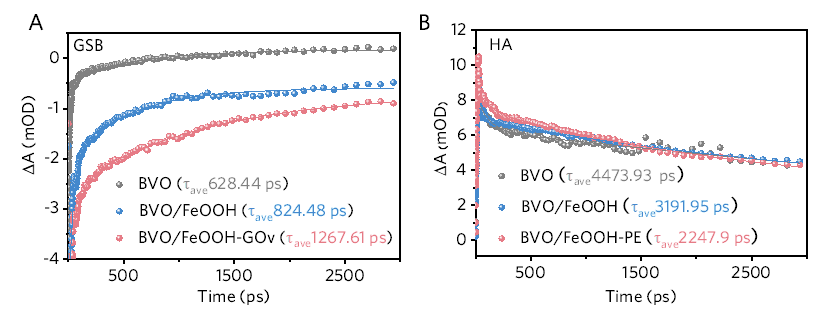


**Fig.** **S26** (**A**) GSB and (**B**) HA signals at selected delay times for BVO, BVO/FeOOH and BVO/FeOOH-GO_V_ films
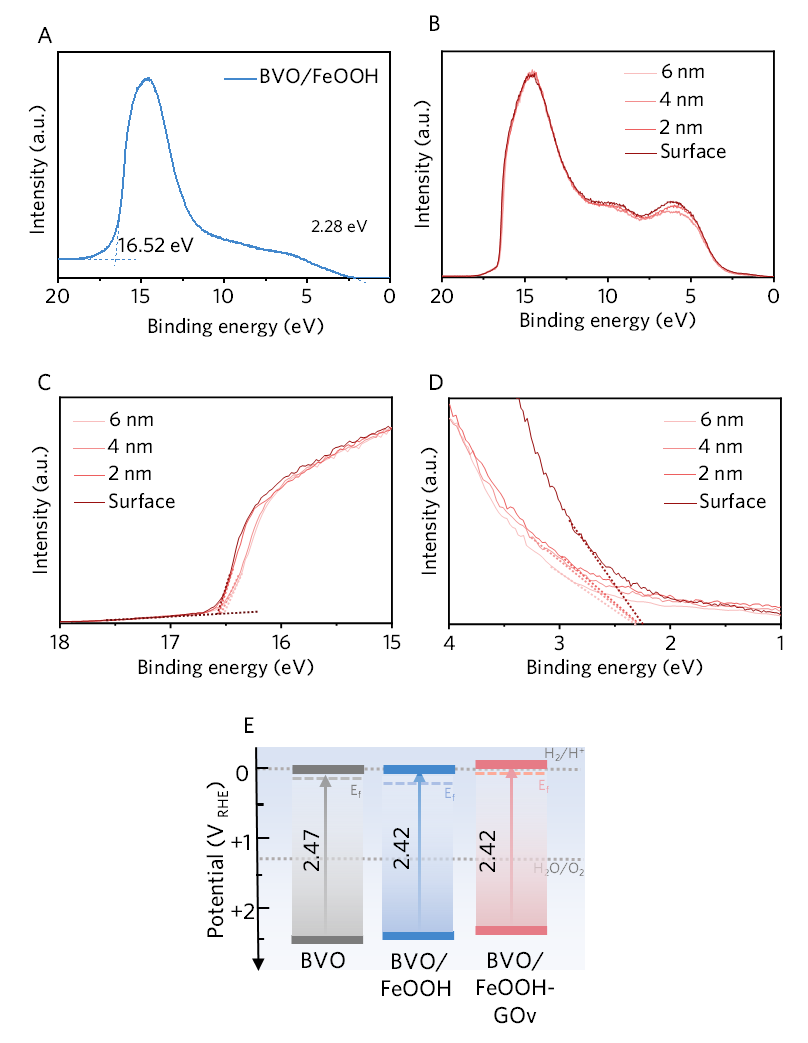


**Fig.** **S27** (**A**) UPS of BVO/FeOOH film. (**B-D**) UPS of BVO/FeOOH-GO_V_ film with different etching depth. (**E**) Band structure of different films


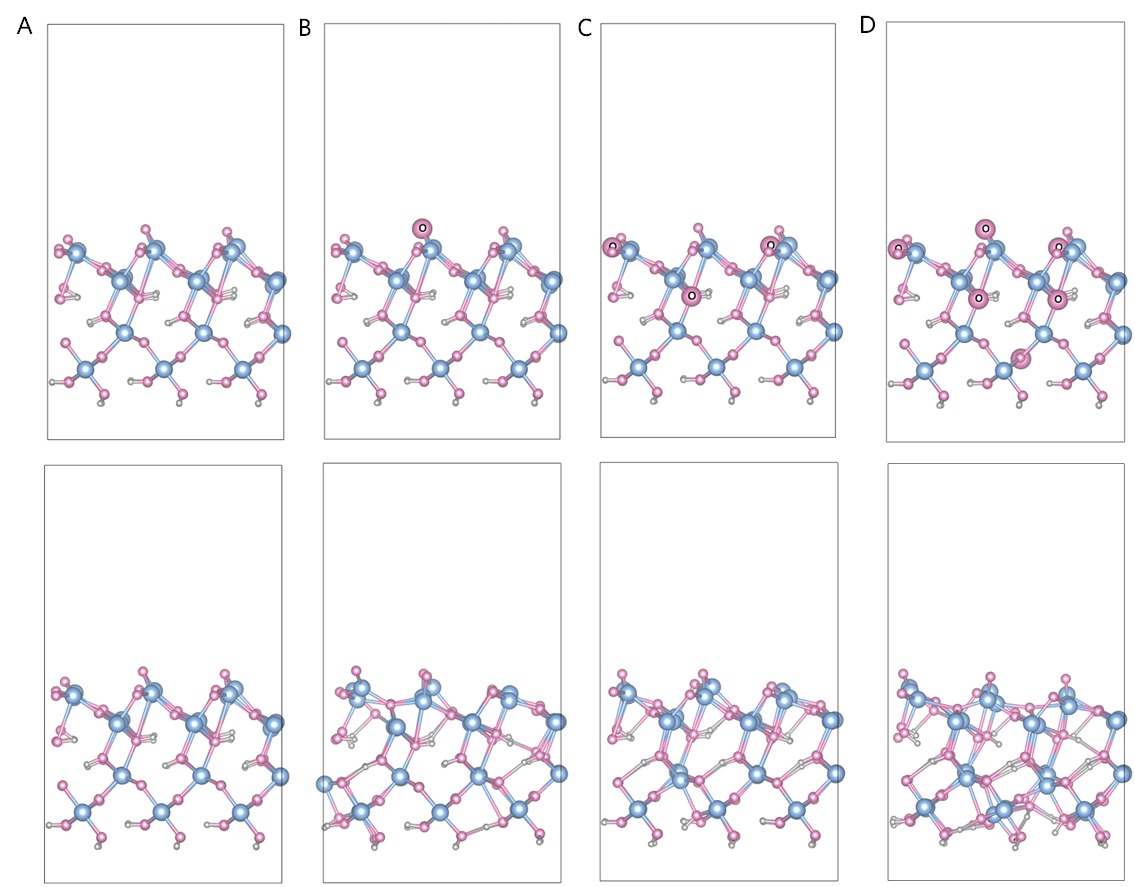


**Fig. S28** The standard orientation of the (**A**) FeOOH, (**B**) FeOOH-GO_V1_, (**C**) FeOOH-GO_V2_ and (**D**) FeOOH-GO_V_ models (first row: initial models; second row: optimized models; bule balls: Fe atoms; pink balls: O atoms; gray balls: H atoms; the larger O atoms marked in the initial models were the deducted O atoms)


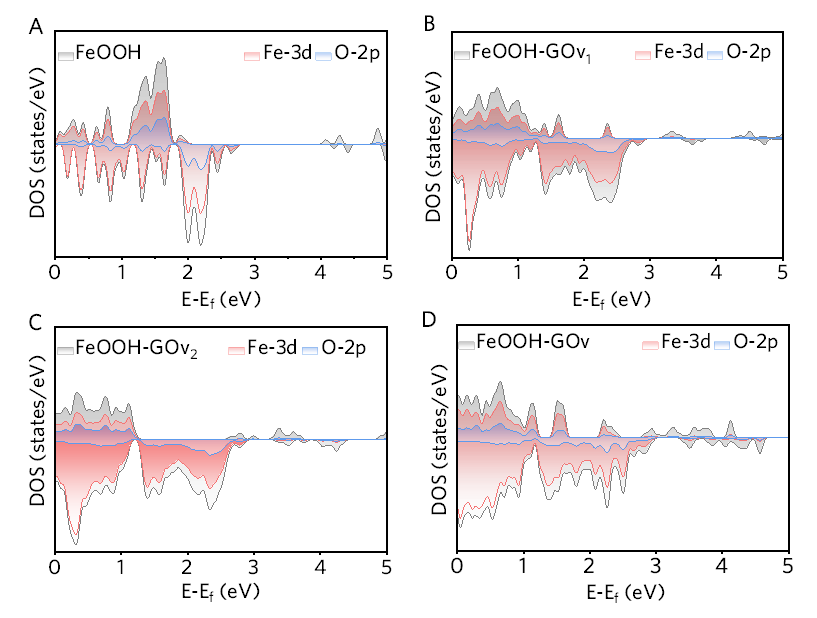


**Fig.** **S29** Enlarged calculated TDOS (gray), Fe-3d (red) and O-2p (bule) PDOS of (**A**) FeOOH, (**B**) FeOOH-GO_V1_, (**C**) FeOOH-GO_V2_ and (**D**) FeOOH-GO_V_, where the Fermi energy level is set to 0


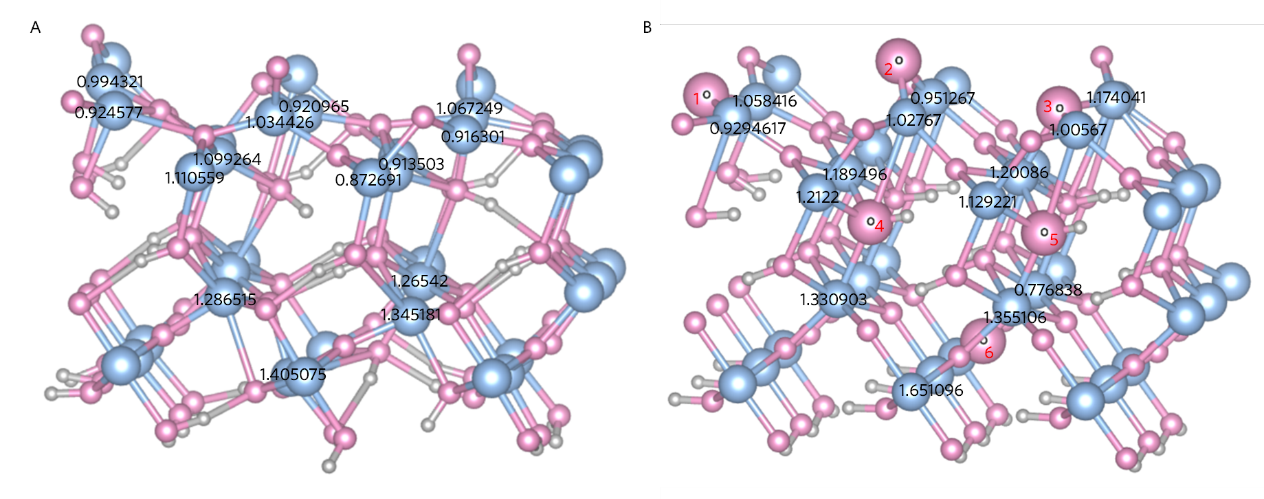


**Fig.** **S30** The Bader charge of Fe atoms nearest to the oxygen vacancies for (**A**) FeOOH-GO_V_ and (**B**) FeOOH model

**Table S1** Recorded values for photocurrent densities and stability from representative FeOOH cocatalysts modified BiVO_4_ films

| Sample | Current density | Stability | Measured at V_RHE_ | Buffer solution | References |
| --- | --- | --- | --- | --- | --- |
| BVO/Ni:FeOOH | 4.15 | 5 h | 1.23 | 1 M KBi (PH~9) | [S14] |
| B-BVO/β-FeOOH | 4.96 | 20 h | 1.23 | 1 M KBi (PH~9) | [S15] |
| BVO /FeOOH(amorphous) | 3.33 | 3h | 1.23 | 0.1 M KHCO_3_ solution (pH 9) | [S16] |
| BVO/β-FeOOH | 4.3 | 2 h | 1.23 | 0.2M Na_2_SO_4_ | [S17] |
| BVO/β-FeOOH | 4.5 | - | 1.23 | 0.2 M Na_2_SO_4_ | [S17] |
| BVO/β-FeOOH | 5.2 | - | 1.23 | 0.2 M Na_2_SO_4_ | [S17] |
| BVO/FeOOH/CQDs | 2.53 | 2 h | 1.23 | 0.2 M Na_2_SO_4_ containing 0.5 M Na_2_SO_3_ | [S18] |
| Ni:FeOOH-coated WO_3_/BiVO_4_ | 4.5 | 3h  (0.8 V_RHE_) | 1.23 | 0.5 M potassium phosphate buffer solution (pH = 7) | [S19] |
| FeOOH/Mo:BiVO_4_ | 3.5 | 6h | 1.23 | 1M phosphate buffer at pH 6.8 | [S20] |
| FeOOH/Au/BiVO_4_ | 4.64 | 2.2h | 1.23 | aqueous solution of 0.1 M Na2SO4 (pH =7) | [S11] |
| FeOOH/In-BiVO_4_(L) | 5.02 | 8h (0.8V_RHE_) | 1.23 | 0.5 M KBi (pH = 9.5) | [S21] |
| BiVO_4_/S-FeOOH | 3.58 | 2h | 1.23 | 0.5 M Na_2_SO_4_ solution | [S22] |
| Vo-BiVO_4_/FeOOH | 4.71 | 11 h | 1.23 | 0.5 M borate buffer (pH = 9.5) | [S23] |
| F:FeOOH/BiVO_4_ | 2.7 | 3 h | 1.23 | 0.5 M Na2SO4 solution (pH = 7.35) | [S24] |
| FeOOH/Ti:BiVO_4_ | 3.99 | 2 h | 1.23 | 0.2 M Na_2_SO_4_ | [S25] |
| Ni:FeOOH/BiVO_4_ | 4.21 | 12 h | 1.23 | 0.2 M  Na_2_SO_4_ | [S26] |
| FeOOH@1T-MoS2@BiVO_4_ | 4.02 | 8 h | 1.23 | 0.1 M potassium phosphate (KH_2_PO_4_) buffer solution | [S27] |
| α-FeOOH(P-II)/BiVO_4_ | 2.64 | 20 h | 1.23 | 0.2 M Na_2_SO_4_ (pH = 7) | [S28] |
| BiVO_4_@Ni:FeOOH | 2.86 | 2h | 1.23 | 0.5M Na_2_SO_4_ electrolyte solution (pH 6.8) | [S29] |
| BVO/FeOOH-GO_V_ | 5.37 | 160h | 1.23 | 1 M KPi | This work |

**Table S2** XPS deconvolution results of O1s spectrum of the pristine BVO, BVO/FeOOH and BVO/FeOOH-GO_V_ photoanodes

| Sample | O_Fe-O_ (%)  529.90 eV | O_OH_ (%)  531.08 eV | O_V_ (%)  531.76 eV | O_A_ (%)  532.57 eV |
| --- | --- | --- | --- | --- |
| BVO | 78.30 | - | 17.13 | 4.57 |
| BVO/FeOOH | 36.12 | 34.07 | 15.54 | 14.47 |
| BVO/FeOOH-GO_V_ | 33.84 | 29.25 | 19.99 | 16.92 |

**Table S3** XPS deconvolution results of Fe 2p spectrum of the BVO/FeOOH and BVO/FeOOH-GO_V_ photoanodes

| Sample | Fe^2+^ (%)  2p 3/2 | Fe^3+^ (%)  2p 3/2 | Fe^2+^ (%)  2p 1/2 | Fe^3+^ (%)  2p 1/2 |
| --- | --- | --- | --- | --- |
| BVO/FeOOH | 41.3 | 58.7 | 41.5 | 58.5 |
| BVO/FeOOH-GO_V_ | 46.3 | 53.7 | 45.9 | 54.1 |

**Table S4** Depth profile deconvolution results of O1s spectrum of the pristine BVO, BVO/FeOOH and BVO/FeOOH-GO_V_ photoanodes

| Etch depth | O_Fe-O_ (%)  529.90 eV | O_OH_ (%) 531.08 eV | O_V_ (%)  531.76 eV | O_A_ (%)  532.57 eV |
| --- | --- | --- | --- | --- |
| Surface | 33.84 | 29.25 | 19.99 | 16.92 |
| 2 nm | 37.20 | 29.09 | 18.20 | 15.51 |
| 4 nm | 37.82 | 31.31 | 16.77 | 14.10 |
| 6 nm | 38.75 | 31.22 | 15.59 | 14.44 |

**Table S5** Band gap (E_g_), VBM (VB) and CBM (CB) positions of BVO and FeOOH

| Sample | E_g_ | VB (V_RHE_) | CB (V_RHE_) |
| --- | --- | --- | --- |
| FeOOH | 2.14 | 2.15 | 0.01 |
| BVO | 2.47 | 2.51 | 0.04 |

**Table S6** Series resistance (Rs) and charge transfer resistance (Rct) of the pristine BVO, BVO/FeOOH and BVO/FeOOH-GO_V_ photoanodes

| Sample | R_s_ (ohm) | R_ct_ (ohm) |
| --- | --- | --- |
| BVO | 52 | 6645 |
| BVO/FeOOH | 55 | 1054 |
| BVO/FeOOH-GOv | 53 | 826 |

**Table S7** Flat band potential (E_fb_) and carrier density (N_A_) of the pristine BVO, BVO/FeOOH and BVO/FeOOH-GO_V_ photoanodes

| Sample | φ_fb_ (V_RHE_) | N_A_ (cm^-3^) |
| --- | --- | --- |
| BVO | 0.37 | 2.02×10^18^ |
| BVO/FeOOH | 0.30 | 5.78×10^18^ |
| BVO/FeOOH-GO_V_ | 0.29 | 8.60×10^18^ |

**Table S8** Comparison of PEC performance at 1.23 V_RHE_ of BVO/FeOOH photoanode in this work with other reprehensive reports on FeOOH-based photoanodes modified by O_V_

| Sample | Current density | Stability | Buffer solution | References |
| --- | --- | --- | --- | --- |
| BVO/Ni:FeOOH  (doping) | 4.15 | 5 h | 1 M KBi (PH~9) | [S14] |
| B-BVO/β-FeOOH  (crystallization) | 4.96 | 20 h | 1 M KBi (PH~9) | [S15] |
| BVO/citrate/FeOOH  (bridging agent) | 3.33 | 3h | 0.1 M KHCO_3_ solution (pH 9) | [S16] |
| BVO/β-FeOOH  (crystallization) | 4.3 | 2 h | 0.2M Na_2_SO_4_ | [S17] |
| BVO/β-FeOOH  (NaBH_4_ reduction) | 4.5 | - | 0.2 M Na_2_SO_4_ | [S17] |
| BVO/β-FeOOH  (Ar-plasma treatment) | 5.2 | - | 0.2 M Na_2_SO_4_ | [S17] |
| BVO/FeOOH/CQDs  (Heterogeneous engineering) | 2.53 | 2 h | 0.2 M Na_2_SO_4_ containing 0.5 M Na_2_SO_3_ | [S18] |
| BVO/FeOOH-GOv  (Photoetching) | 5.37 | 160h | 1 M KPi | This work |

**Table S9** The fit results for the decays at ~443 nm calculated with three exponential decay model of the pristine BVO, BVO/FeOOH and BVO/FeOOH-GO_V_ photoanodes

| Sample | A_1_ | τ_1_ | A_2_ | τ_2_ | A_3_ | τ_3_ | τ_av_ |
| --- | --- | --- | --- | --- | --- | --- | --- |
| BVO | 0.03 | 2.94 | 0.01 | 21.37 | 5.87 | 628.53 | 628.44 |
| BVO/FeOOH | 0.01 | 69.28 | 7.96 | 440.32 | 6.73 | 1020.61 | 824.48 |
| BVO/FeOOH-GO_V_ | 0.01 | 82.49 | 9.31 | 1267.37 | 9.55 | 1267.94 | 1267.61 |

**Table S10** The fit results for the decays ~470 nm calculated with three exponential decay model of the pristine BVO, BVO/FeOOH and BVO/FeOOH-GO_V_ photoanodes

| Sample | A_1_ | τ_1_ | A_2_ | τ_2_ | A_3_ | τ_3_ | τ_av_ |
| --- | --- | --- | --- | --- | --- | --- | --- |
| BVO | 0.49 | 4.16 | 0.41 | 4518.57 | 0.16 | 105.28 | 4473.93 |
| BVO/FeOOH | 0.22 | 47.03 | 0.21 | 3214.62 | 0.21 | 3217.47 | 3191.95 |
| BVO/FeOOH-GO_V_ | 0.39 | 4.54 | 0.09 | 98.444 | 0.42 | 2272.02 | 2247.90 |

**Table S11** The fit results for the decays at ~510 nm calculated with three exponential decay model of BVO/FeOOH-GO_V_ and BVO/FeOOH films

| Sample | A_1_ | τ_1_ | A_2_ | τ_2_ | A_3_ | τ_3_ | τ_av_ | |
| --- | --- | --- | --- | --- | --- | --- | --- | --- |
| BVO/FeOOH | 0.15 | 58.24 | 0.19 | 5962.54 | 0.2 | 5975.83 | | 5947.26 |
| BVO/FeOOH-GO_V_ | 0.18 | 46.6 | 0.25 | 9167.21 | 0.25 | 9160.07 | | 9146.98 |

**Table S12** VBM (VB) and Fermi levels (E_f_) of BVO/FeOOH-GO_V_ films

| Sample | Etching depth | VB (V_RHE_) | E_f_ (V_RHE_) | CB (V_RHE_) |
| --- | --- | --- | --- | --- |
| BVO/FeOOH-GO_V_ | 6 nm | 2.48 | 0.20 | 0.06 |
|  | 4 nm | 2.46 | 0.13 | 0.04 |
|  | 2 nm | 2.43 | 0.11 | 0.01 |
|  | Surface (0 nm) | 2.42 | 0.08 | 0 |
| BVO/FeOOH | - | 2.47 | 0.19 | 0.05 |
| BVO | - | 2.51 | 0.1 | 0.04 |

**Table S13** Bandgap (E_g_) and positions (distance from the vacuum level) of VBM (VB) and CBM (CB) of FeOOH, FeOOH-GO_V1_, FeOOH-GO_V2_ and FeOOH-GO_V_ determined by DFT analysis (E_f_ as 0 eV)

| Sample | Etching depth | VB (V_RHE_) | E_g_ (V_RHE_) | CB (V_RHE_) |
| --- | --- | --- | --- | --- |
| FeOOH-Bulk | 6 nm | 2.80 | 1.96 | 3.96 |
| FeOOH-GO_V1_ | 4 nm | 3.85 | 0.40 | 4.25 |
| FeOOH-GO_V2_ | 2 nm | 4.48 | 0.37 | 4.85 |
| FeOOH-GO_V_ | Surface  (0 nm) | 4.68 | 0.37 | 5.05 |

**Table S14** The average Bader charge of Fe atoms nearest to the oxygen vacancies for FeOOH-GO_V_ and FeOOH model

| FeOOH-GO_V_ | | | | FeOOH | | | |
| --- | --- | --- | --- | --- | --- | --- | --- |
| position | average charge (\|e\|) | layer | average charge (\|e\|) | position | average charge (\|e\|) | layer | average charge (\|e\|) |
| O1 | 0.959449 | First | 0.9676095 | O1 | 0.993938 | First | 1.0244205 |
| O2 | 0.977695 |  |  | O2 | 0.989468 |  |  |
| O3 | 0.965684 |  |  | O3 | 1.089855 |  |  |
| O4 | 1.165446 | Second | 1.132322 | O4 | 1.244192 | Second | 1.179848 |
| O5 | 1.099198 |  |  | O5 | 1.115506 |  |  |
| O6 | 1.338558 | Third | 1.338558 | O6 | 1.261012 | Third | 1.261012 |

Noting: The positions of oxygen vacancies are labeled as O1, O2…, etc.

**Supplementary References**

1. T. W. Kim,K.-S. Choi, Nanoporous BiVO_4_ Photoanodes with Dual-Layer Oxygen Evolution Catalysts for Solar Water Splitting. Science **343**(6174), 990-994 (2014). <http://doi.org/10.1126/science.1246913>
2. X. Zhang, P. Zhai, Y. Zhang, Y. Wu, C. Wang, et al., Engineering Single-Atomic Ni-N_4_-O Sites on Semiconductor Photoanodes for High-Performance Photoelectrochemical Water Splitting. J. Am. Chem. Soc. **143**(49), 20657-20669 (2021). <http://doi.org/10.1021/jacs.1c07391>
3. J. Jian, Y. Xu, X. Yang, W. Liu, M. Fu, et al., Embedding laser generated nanocrystals in BiVO_4_ photoanode for efficient photoelectrochemical water splitting. Nat. Commun. **10**(1), 2609 (2019). <http://doi.org/10.1038/s41467-019-10543-z>
4. Y. Ma, S. R. Pendlebury, A. Reynal, F. Le Formal,J. R. Durrant, Dynamics of photogenerated holes in undoped BiVO_4_ photoanodes for solar water oxidation. Chem Sci **5**(8), 2964-2973 (2014). <http://doi.org/10.1039/c4sc00469h>
5. C. Zachaus, F. F. Abdi, L. M. Peter,R. van de Krol, Photocurrent of BiVO_4_ is limited by surface recombination, not surface catalysis. Chem Sci **8**(5), 3712-3719 (2017). <http://doi.org/10.1039/c7sc00363c>
6. J. F. G. Kresse, Efficiency of ab-initio total energy calculations for metals and semiconductors using a plane-wave basis set. Comput. Mater. **6**, 15-50 (1996).
7. J. A. Stefan Grimme, Stephan Ehrlich, Helge Krieg, A consistent and accurate ab initio parametrization of density functional dispersion correction (DFT-D) for the 94 elements H-Pu. J. chem. phys. **132**, 154104 (2010). <http://doi.org/10.1063/1.3382344>兴
8. S. Feng, T. Wang, B. Liu, C. Hu, L. Li, et al., Enriched surface oxygen vacancies of photoanodes by photoetching with enhanced charge eeparation. Angew. Chem. Int. Ed. **59**(5), 2044-2048 (2020). <http://doi.org/10.1002/anie.201913295>
9. A. N. Ren-De Sun, A. Fujishima, T. Watanabe, K. Hashimoto, Photoinduced Surface Wettability Conversion of ZnO and TiO_2_ Thin Films. J. Phys. Chem. B **105**, 1984-1990 (2001). https://doi.org/10.1021/jp002525j
10. B. Zhang, S. Yu, Y. Dai, X. Huang, L. Chou, et al., Nitrogen-incorporation activates NiFeO_x_ catalysts for efficiently boosting oxygen evolution activity and stability of BiVO_4_ photoanodes. Nat. Commun. **12**(1), 6969 (2021). <http://doi.org/10.1038/s41467-021-27299-0>
11. H. Geng, P. Ying, Y. Zhao,X. Gu, Cactus shaped FeOOH/Au/BiVO_4_ photoanodes for efficient photoelectrochemical water splitting. Int. J. Hydrogen Energy **46**(71), 35280-35289 (2021). <http://doi.org/10.1016/j.ijhydene.2021.08.067>
12. A. Kahraman, M. B. Vishlaghi, I. Baylam, A. Sennaroglu, S. Kaya, Roles of Charge Carriers in the Excited State Dynamics of BiVO_4_ Photoanodes. J. Phys. Chem. C **123**(47), 28576-28583 (2019). <http://doi.org/10.1021/acs.jpcc.9b07391>
13. J. Ravensbergen, F. F. Abdi, J. H. van Santen, R. N. Frese, B. Dam, et al., Unraveling the Carrier Dynamics of BiVO_4_: A Femtosecond to Microsecond Transient Absorption Study. J. Phys. Chem. C **118**(48), 27793-27800 (2014). <http://doi.org/10.1021/jp509930s>
14. M. A. Gaikwad, U. V. Ghorpade, U. P. Suryawanshi, P. V. Kumar, S. Jang, et al., Rapid Synthesis of Ultrathin Ni:FeOOH with In Situ-Induced Oxygen Vacancies for Enhanced Water Oxidation Activity and Stability of BiVO_4_ Photoanodes. ACS Appl. Mater. Interfaces **15**(17), 21123-21133 (2023). <http://doi.org/10.1021/acsami.3c01877>
15. Z. Kang, X. Lv, Z. Sun, S. Wang, Y.-Z. Zheng, et al., Borate and iron hydroxide co-modified BiVO_4_ photoanodes for high-performance photoelectrochemical water oxidation. Chem. Eng. J. **421**(129819 (2021). <http://doi.org/10.1016/j.cej.2021.129819>
16. X. Xiong, C. Zhang, X. Zhang, L. Fan, L. Zhou, et al., Uniformly citrate-assisted deposition of small-sized FeOOH on BiVO_4_ photoanode for efficient solar water oxidation. Electrochim. Acta **389**(138795 (2021). <http://doi.org/10.1016/j.electacta.2021.138795>
17. B. Zhang, L. Wang, Y. Zhang, Y. Ding,Y. Bi, Ultrathin FeOOH Nanolayers with Abundant Oxygen Vacancies on BiVO_4_ Photoanodes for Efficient Water Oxidation. Angew. Chem. Int. Ed. **57**(8), 2248-2252 (2018). <http://doi.org/10.1002/anie.201712499>
18. T. Zhou, S. Chen, J. Wang, Y. Zhang, J. Li, et al., Dramatically enhanced solar-driven water splitting of BiVO_4_ photoanode via strengthening hole transfer and light harvesting by co-modification of CQDs and ultrathin β-FeOOH layers. Chem. Eng. J. **403**, 126350 (2021). <http://doi.org/10.1016/j.cej.2020.126350>
19. L. Cai, J. Zhao, H. Li, J. Park, I. S. Cho, et al., One-Step Hydrothermal Deposition of Ni:FeOOH onto Photoanodes for Enhanced Water Oxidation. ACS Energy Lett. **1**(3), 624-632 (2016). <http://doi.org/10.1021/acsenergylett.6b00303>
20. L. Chen, F. M. Toma, J. K. Cooper, A. Lyon, Y. Lin, et al., Mo-Doped BiVO_4_ Photoanodes Synthesized by Reactive Sputtering. ChemSusChem **8**(6), 1066-1071 (2015). <http://doi.org/10.1002/cssc.201402984>
21. Y. Guo, Y. Wu, Z. Wang, D. Dai, X. Liu, et al., Multi-strategy preparation of BiVO_4_ photoanode with abundant oxygen vacancies for efficient water oxidation. Appl. Surf. Sci. **614**(156164 (2023). <http://doi.org/10.1016/j.apsusc.2022.156164>
22. Y. He, R. Zhang, Z. Wang, H. Ye, H. Zhao, et al., Unveiling the Influence of Sulfur Doping on Photoelectrochemical Performance in BiVO_4_/FeOOH Heterostructures. Anal. Chem. **96**(1), 110-116 (2023). <http://doi.org/10.1021/acs.analchem.3c03287>
23. X. Lu, K.-h. Ye, S. Zhang, J. Zhang, J. Yang, et al., Amorphous type FeOOH modified defective BiVO_4_ photoanodes for photoelectrochemical water oxidation. Chem. Eng. J. **428**, 131027 (2022). <http://doi.org/10.1016/j.cej.2021.131027>
24. H. She, P. Yue, J. Huang, L. Wang,Q. Wang, One-step hydrothermal deposition of F:FeOOH onto BiVO_4_ photoanode for enhanced water oxidation. Chem. Eng. J. **392**, 123703 (2020). <http://doi.org/10.1016/j.cej.2019.123703>
25. J. Wang, J. Bai, Y. Zhang, L. Li, C. Zhou, et al., Unconventional Substitution for BiVO_4_ to Enhance Photoelectrocatalytic Performance by Accelerating Polaron Hopping. ACS Appl. Mater. Interfaces **15**(11), 14359-14368 (2023). <http://doi.org/10.1021/acsami.2c23169>
26. J. Wang, Y. Zhang, J. Bai, J. Li, C. Zhou, et al., Ni doped amorphous FeOOH layer as ultrafast hole transfer channel for enhanced PEC performance of BiVO_4_. J. Colloid Interface Sci. **644**, 509-518 (2023). <http://doi.org/10.1016/j.jcis.2023.03.162>
27. L. Yu, K. Xue, H. Luo, C. Liu, H. Liu, et al., Phase engineering of 1 T-MoS_2_ on BiVO_4_ photoanode with p-n Junctions: Establishing high speed charges transport channels for efficient photoelectrochemical water splitting. Chem. Eng. J. **472**, 144965 (2023). <http://doi.org/10.1016/j.cej.2023.144965>
28. W. Zhang, J. Ma, L. Xiong, H.-Y. Jiang,J. Tang, Well-Crystallized α-FeOOH Cocatalysts Modified BiVO_4_ Photoanodes for Efficient and Stable Photoelectrochemical Water Splitting. ACS Appl. Energy Mater. **3**(6), 5927-5936 (2020). <http://doi.org/10.1021/acsaem.0c00834>
29. X. Zhang, H. Li, W. Kong, H. Liu, H. Fan, et al., Reducing the surface recombination during light-driven water oxidation by core-shell BiVO_4_@Ni:FeOOH. Electrochim. Acta **300**, 77-84 (2019). <http://doi.org/10.1016/j.electacta.2019.01.073>
